# An In Silico Analysis of Malaria Pre-Erythrocytic-Stage Antigens Interpreting Worldwide Genetic Data to Suggest Vaccine Candidate Variants and Epitopes

**DOI:** 10.3390/microorganisms10061090

**Published:** 2022-05-25

**Authors:** Amed Ouattara, Ankit Dwivedi, Matthew Adams, Amadou Niangaly, Matthew B. Laurens, Myaing M. Nyunt, Christopher V. Plowe, Abdoulaye Djimde, Shannon Takala-Harrison, Joana C. Silva

**Affiliations:** 1 Malaria Research Program, Center for Vaccine Development and Global Heath, School of Medicine, University of Maryland School of Medicine, 685 West Baltimore St., Baltimore, MD 21201, USA; madams@som.umaryland.edu (M.A.); mlaurens@som.umaryland.edu (M.B.L.); myaingnyunt@gmail.com (M.M.N.); plowe.chris@gmail.com (C.V.P.); stakala@som.umaryland.edu (S.T.-H.); 2 Malaria Research and Training Center, University of Sciences, Techniques and Technologies of Bamako, Bamako P.O. Box 1805, Mali; niangaly@icermali.org (A.N.); adjimde@icermali.org (A.D.); 3 Institute for Genome Sciences, University of Maryland School of Medicine, 670 West Baltimore St., Baltimore, MD 21201, USA; adwivedi@som.umaryland.edu (A.D.); jcsilva@som.umaryland.edu (J.C.S.)

**Keywords:** plasmodium, liver-stage, antigen, polymorphism, variants, global diversity, multivalent vaccine

## Abstract

Failure to account for genetic diversity of antigens during vaccine design may lead to vaccine escape. To evaluate the vaccine escape potential of antigens used in vaccines currently in development or clinical testing, we surveyed the genetic diversity, measured population differentiation, and performed in silico prediction and analysis of T-cell epitopes of ten such *Plasmodium falciparum* pre-erythrocytic-stage antigens using whole-genome sequence data from 1010 field isolates. Of these, 699 were collected in Africa (Burkina Faso, Cameroon, Guinea, Kenya, Malawi, Mali, and Tanzania), 69 in South America (Brazil, Colombia, French Guiana, and Peru), 59 in Oceania (Papua New Guinea), and 183 in Asia (Cambodia, Myanmar, and Thailand). Antigens surveyed include cell-traversal protein for ookinetes and sporozoites, circumsporozoite protein, liver-stage antigens 1 and 3, sporozoite surface proteins P36 and P52, sporozoite asparagine-rich protein-1, sporozoite microneme protein essential for cell traversal-2, and upregulated-in-infectious-sporozoite 3 and 4 proteins. The analyses showed that a limited number of these protein variants, when combined, would be representative of worldwide parasite populations. Moreover, predicted T-cell epitopes were identified that could be further explored for immunogenicity and protective efficacy. Findings can inform the rational design of a multivalent malaria vaccine.

## 1. Introduction

Malaria eradication will require powerful new tools that interrupt transmission [1,2,3]. Vaccination is the control measure with the greatest impact on past disease eradication efforts [4,5]. Selecting vaccine candidate variants based on laboratory strains has not been a successful strategy for designing malaria vaccines with broad efficacy [6]. Phase-2 trials of a *Plasmodium falciparum* apical membrane antigen 1 (AMA1)-based malaria vaccine [7,8], and a phase-3 trial of the RTS,S vaccine [9] have shown that vaccine efficacy is highest against homologous strains and lower against heterologous strains. This limits the overall efficacy of these subunit vaccines in endemic areas. These findings highlight the fact that the variability of malaria antigens, particularly in epitopes, represents an obstacle for development of a broadly efficacious vaccine, as has been observed with vaccines against pneumococcus [10,11,12] and human immunodeficiency virus (HIV) [13,14].

Over the past decade, genetic and genomic studies have been conducted to assess the diversity of *Plasmodium falciparum* antigens [15]. Most of these studies were limited to specific countries [16,17,18], regions [19,20], or continents [21]. Moreover, these molecular and genomic epidemiology studies were limited to a few antigens mostly present in the blood stage and involved relatively small sample sizes. A further limitation of previous studies was the lack of resolution to epitope-level diversity. We hypothesized that looking at genetic diversity of predicted epitopes may be a more informative option than overall genetic diversity within a given antigen, which may be confounded by immunologically neutral or by highly conserved protein regions that may mask the genetic diversity signal of epitopes. To address all these limitations, we aimed to assess the distribution of global diversity in ten major liver- and sporozoite-stage antigens included in the formulation of vaccines in the development pipeline. Antibodies to the cell-traversal protein for ookinetes and sporozoites (CelTOS, PF3D7_1216600) [22], the circumsporozoite protein (CSP, PF3D7_0304600) [23], and the liver-stage antigen-1 and 3 (LSA1, PF3D7_1036400; LSA3, PF3D7_0220000) [24,25] have all been associated with protection from clinical malaria in humans and other vertebrates. In addition, deletion of the genes encoding P36 (PF3D7_040440) [26], P52 (PF3D7_0404500), [27] upregulated-in-infectious-sporozoites 3 (UIS3, PF3D7_1302200), [28] and upregulated-in-infectious-sporozoites 4 (UIS4, PF3D7_1016900) [29] proteins have been shown to prevent parasite egress from the liver, inhibiting development of blood-stage infection. Finally, liver-stage infections have been prevented by deletion of the sporozoite asparagine-rich protein-1 (SAP1, PF3D7_1147000) [30], while the sporozoite microneme protein SPECT2 (PF3D7_0408700), as the name suggests, has been shown to be important for cell traversal before hepatocyte invasion [31] (Supplemental Table S1).

Many *P. falciparum* antigens, evolving under selective pressure exerted by the host’s immune system, are known to be very diverse [32,33]. Understanding and characterizing selection pressures acting on protein-encoding genes could help identify variants to include in candidate vaccines with broad protection. On the other hand, geographic structuring as observed at the whole-genome level for *P. falciparum* populations, may prevent the design of a single, global malaria vaccine [34,35], limiting vaccine efficacy to specific regions defined by the geographic distribution of strains similar to the vaccine strain. The public availability of *P. falciparum* whole-genome sequences from around the world opens the door to comprehensive analyses of selection within, and population differentiation between, geographic populations. Inferences from these analyses could facilitate the design of broadly protective, multivariant malaria vaccines. By using whole-genome sequence data and associated metadata on geographic location to screen for candidate antigen variants, this study aimed to better understand each antigen’s natural variation in the context of geographic distribution. By characterizing the extent of genetic diversity in genes that encode liver-stage antigens, we hoped to identify the most frequent alleles and/or conserved epitopes of each protein that may be integrated in the design of broadly protective malaria vaccines.

## 2. Materials and Methods

### 2.1. Antigen Selection

Vaccine candidate antigens selected in this study are in preclinical development or in early-stage clinical trials, excepting CSP, which forms the basis for the RTS,S vaccine, which underwent a large-scale, pilot implementation trial in Africa [36] and has been endorsed by World Health Organization (WHO).

### 2.2. Allelic Sequence Generation

Sequence reads from whole-genome sequencing datasets were aligned to the reference 3D7 genome using Bowtie2 [37]. Base recalibration was conducted with the MalariaGEN polymorphism panel, and single-nucleotide polymorphism (SNP) calls were made using GATK’s UnifiedGenotyper v4.1.8.1 [38,39] with the major allele called for each polymorphic site. The high-confidence SNPs were selected using the filter “DP < 12 || QUAL < 50 || FS > 14.5 || (MQ0 ≥ 2 && (MQ0/(1.0 × DP)) > 0.1)”. For each sample, the nucleotide sequences for the ten antigen-encoding genes were reconstructed by using the 3D7 *P. falciparum* alleles as the backbone on which the SNP calls (synonymous and nonsynonymous) for each sample were introduced, using GATK’s v3.7 FastaAlternateReferenceMaker [38]. The missing genotypes were considered as reference alleles. The sequences were recovered in correct orientation corresponding to the localization of antigens on the specific strand. Sequences from Brazil (*n* = 22), Burkina Faso (*n* = 56), Cambodia (*n* = 129), Colombia (*n* = 16), Cameroon (*n* = 130), French Guiana (*n* = 36), Guinea (*n* = 123), Kenya (*n* = 57), Malawi (*n* = 150), Mali (*n* = 99), Myanmar (*n* = 18), Papua New Guinea (*n* = 59), Peru (*n* = 11), Tanzania (*n* = 68), and Thailand (*n* = 36), as reported in [35] or obtained from MalariaGEN (Parasite genomic surveillance: www.malariagen.net/data/pf3k-5 (accessed on 9 March 2019)) (Supplemental Table S2), were used in the analysis. The analyses did not consider structural and copy number variations and focused only on SNP variants.

### 2.3. Data Analyses

#### 2.3.1. Nucleotide Polymorphism

To assess if the candidate vaccine antigens would evolve under a neutral model, genetic diversity statistics were assessed separately for each sample set, and at the worldwide level, when warranted, using DnaSP [40]. The parameters calculated included the number of polymorphic sites (S), nucleotide diversity per synonymous (*π*_S_) and nonsynonymous (*π*_N_) site, overall nucleotide diversity (*π*), and the number of unique haplotypes for each given antigen.

#### 2.3.2. Haplotype Diversity and Distribution

Haplotypes were based on the whole coding sequence for each gene without their untranslated regions. The frequencies of the three major haplotypes of each antigen were compared between countries and regions using a chi-square test in SAS 9.4^®^. The mean haplotype diversity was compared between countries and regions using the non-parametric, 2-sided Mann–Whitney test. Antigens with three haplotypes representing more than 80% of a country’s parasite population were considered “priority” vaccine candidates. Immunogenic amino acid fragments that are conserved across countries could be ideal cross-protective vaccine candidates. Thus, for each antigen, conserved fragments, defined as a stretch of at least 20 amino acids without polymorphic residues in the 3D7 reference strain, were identified from the worldwide diversity dataset, and variant proportions were compared using a chi-square test in SAS 9.4^®^. This metric could identify short amino acid sequences that could be used in a multifragment malaria vaccine candidate.

Haplotype networks are commonly used to visualize the relationship between haplotypes from different sampling sites. Haplotype networks were generated with sequences both within sampling locations (indicating the unit of geographic sampling, e.g., country, region, continent) and for the global dataset by median-joining network analysis using PopART (Population Analysis with Reticulate Trees) software [41]. The dataset used was based only on nonsynonymous nucleotides (compared to the 3D7 reference), and the between-country, -region, and -continent frequencies of subpopulations were compared using chi-squared tests (SAS 9.4^®^).

#### 2.3.3. Population Structure

To assess the null hypothesis that allele frequencies do not vary between countries, regions, or continents, we computed F_ST_ (gene fasta files), as implemented in DnaSP v6 [42], with significance assessed by permutation of sequences among geographic locations, with 10,000 replicates.

#### 2.3.4. Cytotoxic T-Lymphocyte (CTL) Epitope Identification and Polymorphisms

To assess if polymorphisms in candidate genes were located in putative epitopes, we used the T-cell epitope prediction algorithm (NetMHCpan-3.0, http://www.cbs.dtu.dk/services/NetMHCIIpan-3.0 (accessed on 28 October 2021)) and common human leukocyte antigen (HLA) frequencies in Mali, a country in West Africa with high malaria incidence (HLA-A*23:01, HLA-A*30:01, HLA-B*35:01, DRB1_1304, DRB1_0701, DRB1_1101, DRB1_0120) [43,44] to screen all candidate gene sequences of the reference strain 3D7 for potential CD4+ and CD8+ T-cell epitopes. These HLA types are part of the three supertypes (HLA-A2, -A3, and -B7) [45,46]. A putative epitope cutoff was set with binding strengths of up to 50 nM indicating strong-binding epitopes and strengths from 51 to 500 nM indicating intermediate binders [47]. All epitopes identified through these analyses were used to screen for potential SNPs in the homologous genomic regions in 1010 samples from 15 countries included in this study.

#### 2.3.5. B-Cell Epitopes and Polymorphisms

Structural epitopes are groups of amino acid residues that are antibody targets. Most B-cell epitopes are discontinuous, comprising from 15 to 25 nonsequential residues [48], and these conformational B-cell epitopes represent more than 90% of functional B-cell epitopes [49]. We used CBTOPE to predict conformational B-cell epitopes using the 3D7 strain [50]. The approach uses support vector machines (SVM) to predict discontinuous epitopes with an accuracy greater than 85% and a sensitivity assessed as an area under curve (AUC) of 0.9. Following B-cell epitope residue prediction, we concatenated discontinuous residues into single fragments (epitope fragment) that define individual samples and assessed their polymorphism in the complete dataset. Thus, each sample was defined by its polymorphic residues within regions orthologous to predicted B-cell epitopes in the reference 3D7. The frequencies of these epitope fragments were compared between countries and regions using a chi-square test in SAS 9.4^®^.

#### 2.3.6. Polymorphisms in Epitopes and Protein Function

We used the PROVEAN (Protein Variation Effect Analyzer) software [51] to identify SNPs predicted to be functionally important. Briefly, sequences are initially clustered based on sequence alignment scores. The scores are averaged within and across clusters to generate the PROVEAN score. If the PROVEAN score is equal to or below a predefined threshold of −2.5, the SNP(s) within the gene’s coding sequence is(are) predicted to have a “deleterious” effect on protein function, while a score above this threshold is equivalent to a “neutral” effect. The −2.5 cutoff has been shown to have 81% sensitivity, 75% specificity, and 75.17% balanced accuracy using UniProt nonhuman protein variant datasets [52].

#### 2.3.7. Secondary Predicted Structure and Solvent Accessibility

The NetSurfP 2.0 server (https://services.healthtech.dtu.dk/service.php?NetSurfP-2.0 (accessed on 30 October 2021)) was used to predict each candidate protein’s structural features, including their secondary predicted structure and surface accessibility, using nucleotide sequences in fasta format. The sequence of each gene in the reference 3D7 strain was used in COACH (Consensus Approach) and COACH-D (Consensus Approach 3D) to predict proteins’ structures [53,54]. This approach uses S-SITE [53] and TM-SITE [53] comparative method data combined with results from COFACTOR [55], FINDSITE [56], and ConCavity approaches [57] to predict proteins’ structures and ligand binding sites.

## 3. Results

To identify malaria vaccine candidate variants, or, more specifically, epitopes and protein fragments, that will not be the target of allele-specific efficacy, we surveyed the genetic diversity in the genes encoding ten *P. falciparum* pre-erythrocytic-stage antigens currently in development or in clinical testing. We obtained their DNA and/or protein sequences from 1010 *P. falciparum* whole-genome sequence datasets. These datasets (Supplemental Table S2) (Parasite genomic surveillance: www.malariagen.net/data/pf3k-5 (accessed on 9 March 2019)) were obtained from the public domain (*n* = 721) or generated in-house (*n* = 289). Each of these vaccine candidates was analyzed separately to select potential infection-blocking vaccine candidates that could enter a vaccine development pipeline.

### 3.1. Protein Diversity and Mode of Evolution

Malaria vaccine antigen diversity can negatively impact efficacy in endemic areas [9,58,59,60,61,62]. To identify proteins that are less likely to be susceptible to allele-specific efficacy, we estimated, for each locus, haplotype diversity (*Hd*) and nucleotide diversity (*π*), following the rationale that the proteins with the fewest variants are the least likely to lead to vaccine evasion. While *Hd* estimates the probability that two randomly sampled alleles are different, *π* estimates the average number of nucleotide differences per site between two randomly sampled alleles. The analyses showed that *Hd* estimates ranged from 0.21 for UIS3, the least diverse protein, to 0.99 for the most diverse protein (SAP1), and the *π* statistic for the ten candidate proteins screened ranged from 0.038% for UIS3 to 1.530% for CelTOS (Table 1).

To determine if the genetic diversity observed was similarly represented across geographic regions, we estimated *F*_ST_ between all pairs of geographic regions (Supplemental Table S3). *F*_ST_ statistics estimated using samples from the four regions showed low-to-great differentiation (*F*_ST_ values ranging from 0005 to 0.68) between regions. Moreover, our analyses showed that for each antigen, *F*_ST_ values ranged from a lack of clustering to a complete subdivision at the country level (Supplemental Table S4), with UIS3 protein showing a lack of subdivision between countries (*F*_ST_ values ranging from 0 to 0.14).

To determine the diversity of each protein, we started by estimating haplotype diversity. Most proteins except P36 and UIS3 showed considerable polymorphism suggestive of an excess of rare alleles (Table 1).

To assess whether conserved regions exist in protein-encoding genes, we aligned all the sequences from the 15 countries and identified fragments that were invariable in the whole dataset of 1010 sequences for each antigen. Using a chi-square test for differences of proportions, there was a significant difference in the proportion of conserved fragments (number of conserved fragments divided by the reference protein length) obtained from each antigen (*p* = 0.02) with only 1 fragment identified in UIS4 and 58 fragments in SAP1 (Table 1).

### 3.2. Vaccine Antigen Geographic Distribution and Haplotype Diversity

**Figure 1:**
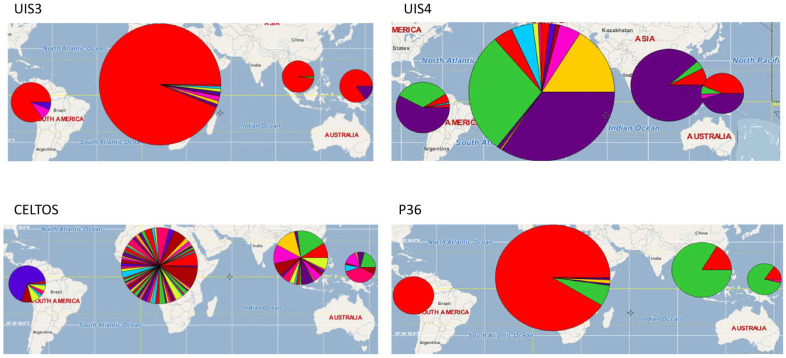
Worldwide distribution of amino acid sequences of four malaria vaccine candidates. Panel A represents haplotypes for upregulated-in-infectious-sporozoites 3 (UIS3), while panel B shows the worldwide distribution of UIS4 sequences. Panel C represents P36 sequences, and panel D is a world map view of the distribution of sequences of the cell-traversal protein for ookinetes and sporozoites (CelTOS). The size of the pie is proportional to the sample size used, and each slice of the pie represents a haplotype with color consistency across panels. CelTOS is the most diverse protein, while P36 is the most conserved. Whereas the major haplotype of UIS3 is consistent across continents, the major haplotype of P36 in Africa is the minor haplotype in other continents.

**Figure 2:**
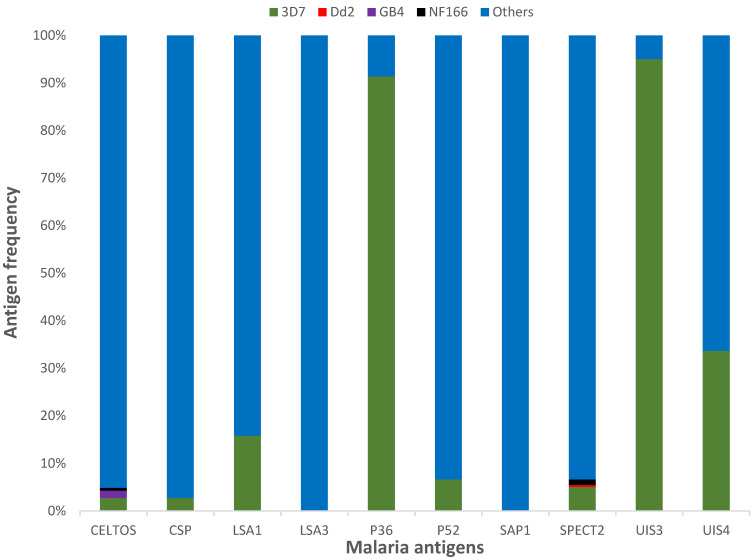
Frequencies of ten liver-stage malaria vaccine candidates. Antigens’ names are listed on the *x* axis, while frequencies are on the *y* axis. The frequencies are stacked to 100%, and each color represents a variant or a group of variants. The laboratory strain 3D7 is represented in green.

Determining if protein variants in each of the candidate vaccine loci are specific to a given country or if, instead, they are distributed worldwide, together with their relative frequency, can provide invaluable information for the design of a malaria vaccine that is cross-protective. To visualize the worldwide distribution of the sequences of each of the most conserved antigens, a haplotype distribution pie chart and a plot (Figure 1 and Figure 2) were built. While moderate substructuring (*F*_ST_ values ranging from 0.05 to 0.15) and higher substructuring (*F*_ST_ values ranging from 0.16 to 0.25) of variants were observed for most antigens, UIS3, UIS4, and P36 had the same predominant haplotype in all continents (Supplemental Table S5). These analyses showed that the greatest polymorphism in UIS3, UIS4, and P36 was observed in Africa, with the predominant variant of each of these antigens representing, respectively, 95.02%, 33.67%, and 91.4% of all variants sampled. Overall, the most frequent antigen variants of UIS3 and UIS4 were the same in all continents, while the major variant of the P36 antigen in Africa and South America was the second most frequent in Asia and Oceania and vice versa (Figure 1).

To identify protein variants that, when combined, could represent a large portion of worldwide diversity within these vaccine candidate proteins, we measured the prevalence of each protein variant at the country, continent, and worldwide levels. While the main variants of most proteins were less than 4% of the global parasite population studied, the worldwide frequencies of the most frequent variant for UIS3, UIS4, and P36 were 96.1%, 46.5%, and 74.1%, respectively (Figure 2), suggesting that a combination of two or three variants of these candidates could cover the global antigenic diversity. Overall, seven out of the ten proteins were highly diverse (Table 1), potentially limiting their effectiveness as malaria vaccine candidate antigens due to the high risk of vaccine-induced selection when deployed in the field, as happened with the pneumococcus vaccine PCV13 [63].

### 3.3. Identification of T-Cell Epitope and Polymorphisms

To identify potential constituents of a multiepitope-based malaria vaccine containing the most conserved sequences, protein regions encoding putative CD4+ and CD8+ T-cell epitopes in the reference 3D7 alleles were characterized for polymorphism using global isolates. These putative epitope sequences were identified using the NetMHCpan-4.1 prediction algorithm and common human leukocyte antigen (HLA) frequencies in Mali [44,64], a centrally located country in West Africa, one of the regions in the world with the most intense malaria transmission. Predicted epitopes were distributed across all proteins (Supplemental Table S6), and a multiple sequence alignment of these regions was used to identify SNPs located in each epitope. Results show a great variability in the average number (sum of CD4+ and CD8+ epitopes/ number of amino acids) of medium- and strong-binding T-cell epitopes in each protein (*p* < 0.001). While P36 had the highest average number of CD4+ and CD8+ linear T-cell epitopes (9.3), few CD4+ and CD8+ (1.4) epitopes were predicted in LSA1 (Supplemental Table S6). CelTOS and P36 had completely conserved CD4+ and CD8+ epitopes across the sequences used in this study. CSP, LS1, P36, and P52 had no polymorphisms in their CD4+ T-cell epitopes, but some variation was detected in the CD8+ epitopes of these proteins. CD8+ epitopes in SAP1, SPECT2, and UIS3 were invariant in the whole dataset. Finally, UIS4 had only one CD4+ epitope with mutations. Moreover, UIS3 and UIS4 had no detectable CD8+ epitopes at the medium and strong binder cutoff values used and the HLA alleles investigated. SAP1 and LS3 had no CD4+ epitopes that were strong or medium binders, again with the same potential caveats regarding cutoff values and HLA genotypes.

Overall, for each antigen, we detected significant differences in the number of epitopes that could be used as components of a multiepitope malaria vaccine, with CelTOS and P36 having both CD4+ and CD8+ conserved T-cell epitopes.

### 3.4. B-Cell Conformational Epitopes and Polymorphisms

Using the conformational B-cell epitope (CBTOPE) server for conformational epitope prediction and epitope sequence analysis tools, we identified conformational epitopes of all ten candidates and generated a concatenated fragment of individual nucleotides/epitopes. The resulting major epitope-containing fragment of CelTOS represented only 7.03% of the whole dataset sequences, while two and three major epitope-containing fragments of SPECT2 and CSP represented, respectively, 87% and 61.4% of the dataset’s epitope-containing fragments. LSA1 (90%), LSA3 (97%), P36 (99.8%), P52 (99.9%), and SAP1 (90.4%) each had one major epitope-containing fragment, representing at least 85% of the dataset variability. Furthermore, an assessment between countries and continents showed no differences in epitope-containing fragment distribution at the country and continent level (all *p* values greater than 0.05). Although eight out of ten of these antigens had relatively conserved B-cell epitope-containing fragments, reported polymorphic residues were scattered across antigen-encoding genes with polymorphisms located in binding sites of LSA1, LSA3, P52, CelTOS, SAP1, and CSP (Supplemental Figure S1). Interestingly, P36, UIS4, and UIS3 had no polymorphic residues in antibody binding sites.

### 3.5. T-Cell Epitope Biological Relevance and Solvent Accessibility

The impact of amino acid polymorphisms on protein/peptide function was evaluated with PROVEAN (Protein Variation Effect Analyzer) [51,52]. None of the 36 amino acid polymorphisms identified in putative LSA1 epitopes were predicted to have a deleterious effect on protein function. The same findings were obtained for polymorphisms identified in putative epitopes of CSP, LSA3, UIS3, SPECT2, SAP1, P52, and P36. In contrast, PROVEAN predicted that nonsynonymous changes in putative epitopes of UIS4, UIS3, SAP1, and P36 likely have deleterious effects on protein function, suggesting that these epitope-encoding protein regions can tolerate some variation despite potentially negative fitness effects on the parasite, or the proteins may be functionally constrained. Finally, all putative epitopes encoded in the *celtos* gene were conserved, potentially allowing for an immune response to target an epitope whose sequence may be under strong functional constraints.

**Figure 3:**
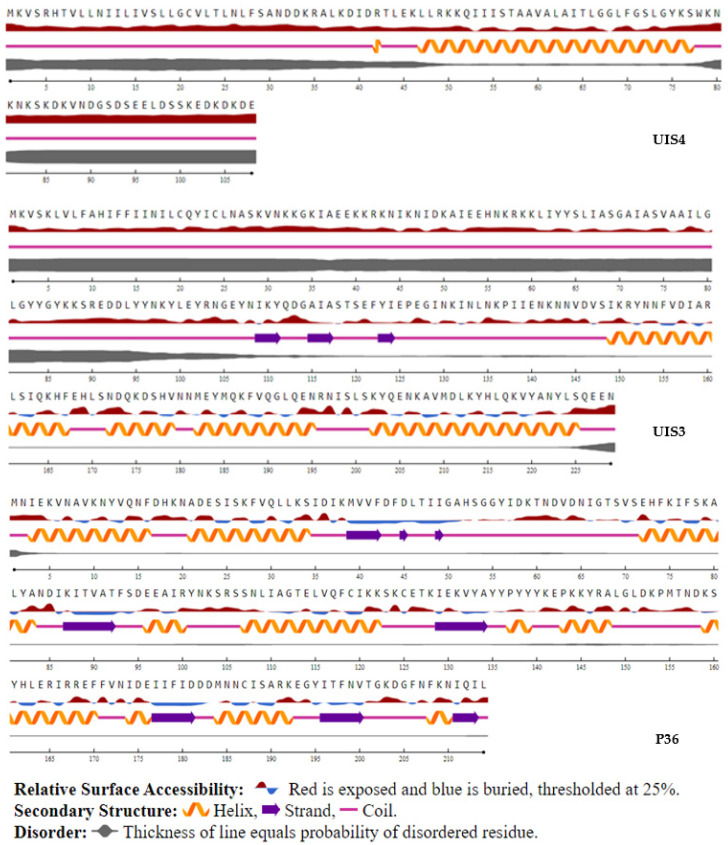
Predicted secondary structure and surface accessibility of P36, UIS3, and UIS4 malaria vaccine candidates.

**Figure 4:**
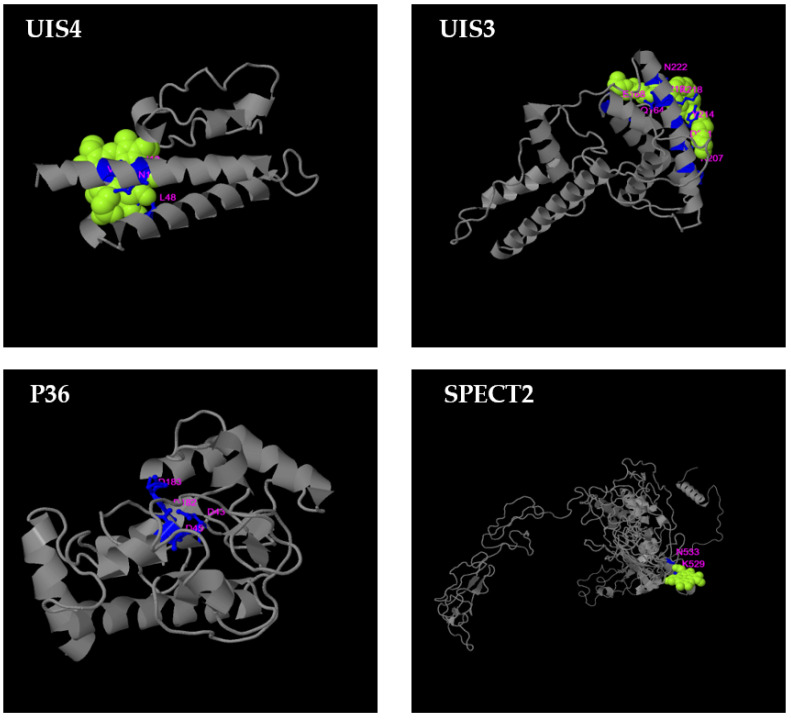
Predicted 3D structure of four malaria vaccine candidates with hypothetical protein–ligand binding sites shown in light purple.

Disordered regions in proteins/peptides (regions that do not fold into a fixed three-dimensional structure), as well as their surface accessibility to the solvent and secondary structure, were predicted using NetSurfP. Except for P36 and P52, disordered regions were distributed across all proteins, while, excepting a few protein fragments, all protein surfaces were accessible to the solvent (Supplemental Figure S2). Our results show that, with a few exceptions, putative epitopes generated from P36 and UIS4 proteins had no deleterious amino acids (Figure 3), were accessible to the solvent, and were mostly not in a helix-forming region (Table 2). Conserved CelTOS, P52, SPECT2, CSP, UIS4, UIS3, LSA1, and LSA3 epitopes were exposed to solvent but were in disordered and/or helix-forming regions (Supplemental Figure S2). Finally, all except for one peptide of the 33 SAP1 conserved peptides were in disordered regions (Supplemental Figure S2). Predicted 3D structure of malaria vaccine candidates were generated using COACH and COACH-D meta servers (Figure 4 and Supplemental Figure S3), followed by an identification of hypothetical protein-ligand binding sites. Probable binding residues [54] were identified based on their C-score, which is a measure of the confidence score of the prediction. All antigens have low-reliable putative binding site(s).

## 4. Discussion

This study characterized the genetic diversity in ten pre-erythrocytic *P. falciparum* antigens that are currently included in the formulation of malaria vaccines in clinical development. Thanks to the public availability of whole-genome data for thousands of *P. falciparum* isolates from around the world [65], it is now possible to conduct genome-wide analyses in silico without the need to generate additional sequence data, especially from geographically distributed regions that represent diverse malaria transmission patterns. Malaria vaccinologists thus have at their disposal tools and information that can be used to inform vaccine design, long before field trials are planned. To generate a baseline understanding of the background genetic variation in promising vaccine antigens, we investigated the geographic distribution of genetic variants of 10 putatively sporozoite-/liver-stage antigens. As some antigens are expressed during both liver and blood stages, we aimed to evaluate their potential effectiveness in preventing infection as well as any potential synergistic role they could play in preventing blood-stage disease [66].

The *P. falciparum* liver stage is an ideal parasite developmental stage for the investigation of potential vaccine targets as efficacy directed against this stage can provide protection against both infection and disease [67]. While the most advanced malaria vaccine candidate [68] is based on CSP, the major protein expressed on the sporozoite surface, CSP diversity [9] may have led, in part, to low efficacy in the field [69,70]. While new immunization approaches aiming to boost cellular and humoral responses are being used to improve RTS,S-induced protection [71,72,73], multiple additional liver-stage antigens are still in the vaccine development pipeline [74,75,76].

We have identified the specific variants of UIS3, UIS4, and P36 that cover worldwide diversity and, if appropriately immunogenic, could be used to formulate a cross-protective malaria vaccine. Furthermore, the lack of polymorphisms in binding and structurally relevant sites within these antigens suggests that potential mutations are highly detrimental to parasite fitness, which may prevent emergence of vaccine-resistant strains [77,78].

Molecular epidemiology and population genetic analyses from a total of 1010 allelic sequences indicated that UIS3 with one variant, P36 with one variant, and UIS4 with three variants represented more than 90% of the worldwide diversity and were the most conserved of the ten proteins analyzed. Importantly, the allele in the 3D7 reference strain, on which in-development vaccines incorporating these antigens are based [27,75,79], was the predominate variant in all countries. Consistent with published reports [20,80,81], our analyses of worldwide samples have shown high nucleotide diversity in African samples compared to Asian, South American, and Oceanic samples. In addition, haplotype diversity and the distribution of the frequencies of major alleles were higher in Africa compared to the rest of the world. Many factors could explain these high frequencies. Although malaria incidence has declined worldwide since 2010, Africa still has the highest burden of the disease [82]. The high transmission intensity [82], the large effective population size of *P. falciparum* [83], and selective pressure due to acquired immunity may explain the high diversity observed in Africa. In addition, as multiplicity of infection is more frequent in Africa compared to other continents [84,85], the probability of mosquitoes carrying multiple alleles is increased, potentiating more intragenic recombination leading to more diversity in the parasite population. However, among the 10 liver-stage antigens evaluated, only UIS3, UIS4, and P36 were conserved across countries and continents, making them ideal for vaccine design.

Additional analyses based on *F*_ST_, haplotype geographic distribution, supported a low-to-moderate subdivision of these three antigens by country and continent. Parallel to our findings, previous studies [80,81] that focused on the erythrocyte stage of parasite development also found subpopulations and substructuring at the country and continent levels. These population subdivisions could be explained by the same immune mechanism happening during the liver and erythrocyte stages. 

Although P36 showed significant differentiation between continents, this substructuring at the continent level was clearly explained by the high prevalence of the 3D7 variant of P36 in Africa compared to Asia and the Americas. However, in Asia and the Americas, the 3D7 variant of P36 was the second most frequent variant, which may allow the design of a bivalent P36-based vaccine that would cover at least 80% of the worldwide parasite population. As studies have shown that polymorphic regions of proteins may be involved in immune escape [60,86,87], the high haplotype diversity and the relatively high abundance of minor variants in seven out of the ten antigen candidates may make it difficult to design a global vaccine based on those candidates.

As the expression of full-length malaria antigens has been challenging [88], the consideration of epitope-based vaccines has become more prevalent [89]. Contributing to the popularity of epitope-based vaccines is the fact that protein expression systems have various limitations when expressing *P. falciparum* proteins. The lack of post-translational modifications in addition to its inefficiency in expressing higher-molecular-weight proteins has limited the use of *Escherichia coli* in malaria vaccinology [90]. Although insect cell and yeast expression systems are attractive for their post-translational modifications and large yield, and mammalian cell expression systems are appealing because of their ability to recognize all protein synthesis signals [90,91], these systems are very expensive, limiting their use in initial antigen screening. Thus, fragment- or epitope-based vaccines comprising UIS3, UIS4, and P36 fragments may be good alternatives to whole proteins, if they can elicit the necessary inhibitory capacity.

CD8+ T cells play a critical role in an effective immune response against pre-erythrocytic-stage malaria parasites [92]. This protection is dependent on CD4+ T cells [93,94] and may also require neutralizing antibodies [95]. Furthermore, studies have shown that polymorphisms in relevant epitopes could alter their binding to receptors and affect protein recognition by the immune system [96]. To identify epitopes that could trigger effective B- and T-cell responses and thus replace whole proteins in a multivalent epitope-based vaccine, we screened each vaccine candidate to select linear T-cell epitopes and linear and conformational B-cell epitopes. The P36 antigen had strong-binding, highly conserved CD4+ and CD8+ epitopes; while the binding strength of UIS3 and UIS4 epitopes was lower than that of P36, they contained few polymorphisms. The selection of high-score B-cell epitopes using CBTOPE showed that P36, UIS3, and UIS4 were the three candidates without polymorphisms in any of the predicted epitopes. Although antibodies may not recognize these targets (lack of antigenicity), the design of a vaccine with immunogenic B- and T-cell epitopes could be based on these three antigens by covalently attaching the candidate protein(s) to an immunogenic carrier. In vitro microarray studies are being conducted to characterize and assess the immunogenicity of these candidates in humans.

Although ex vivo assays are ideal to evaluate the phenotype and function of T cells in peripheral blood along with antibody response, in silico analyses could be used as screening techniques to rule out peptides with a low binding affinity for their targeted ligand and those with polymorphic residues that are detrimental to the receptor and ligand interaction. Hence, binding residues were predicted and polymorphisms that may have an impact on protein binding to its receptors were identified. These analyses indicate P36 was the single antigen with the strongest binding peptides, and the residues of these peptides were conserved across samples from all continents surveyed.

Although a large dataset was used to assess the worldwide diversity of these ten liver-stage candidates, the recent release of Pf7k (www.malariagen.net/pf7k (accessed on 9 March 2019)), which includes more sequences from these countries, may help improve haplotype frequency estimates. Hence, additional analyses are being conducted to validate P36 findings. Major histocompatibility complex (MHC) and HLA [97] polymorphisms are the major hurdles in the prediction of T-cell epitopes [98]. Thus, a limitation of this study was the limited selection of HLA genotypes used, which cannot be considered representative of the worldwide HLA distribution. However, three supertypes, including the ones used in this study, have been shown to cover at least 88% of the worldwide diversity. As the reliability and accuracy of the different prediction algorithms used could only be confirmed by in vitro and/or in vivo testing, we are conducting in vitro invasion inhibition assays and ex vivo microarray assays using serum from infected patients to assess their functional relevance. Finally, it is noteworthy to mention that B-cell epitope prediction is usually less reliable than T-cell epitope prediction [99].

Overall, our findings suggest that among all ten antigens tested, P36, UIS3, and UIS4 were the top candidates for a successful strain-transcending vaccine based on protein residue conservation and the existence of many B- and/or T-cell epitopes. However, P36 stands out as the best candidate for the conservation of its epitopes and the lack of polymorphisms at functionally relevant residue positions. The essential role the protein plays during hepatocyte invasion [27] makes it a good target for a liver-stage vaccine, as a knockout of the gene encoding P36 has led to parasite developmental arrest at the early stage of hepatocyte infection [27]. Since preclinical testing in the mid-2000s [26], new data have not been available on its use in early clinical trials. We have generated full proteins and peptides of P36 to assess its relevance in ex vivo assays and immunogenicity in in vivo analyses.

## 5. Conclusions

We have presented herein findings from in silico analyses for the screening and selection of malaria vaccine candidates. These results indicate that instead of CelTOS, CSP, LSA1, LSA3, SPECT2, or SAP1, a few variants of P36, UIS3, or UIS4 could be used to cover the worldwide diversity of these proteins and, were they to induce a protective response, prevent vaccine escape when the vaccine is deployed in the field. Moreover, epitope prediction and analysis, in addition to ongoing functional assays, will validate the use of these approaches in epitope-based vaccine design. These approaches applied systematically may accelerate malaria vaccine discovery.

## Figures and Tables

**Table 1:** Genetic diversity parameters of 10 infection-blocking vaccine candidates.

Antigens	*Hd*	π Per 100 bp	Breaks in Sequence Conservation across Haplotypes
SAP1	0.9990	0.181	58/2940
LSA3	0.9989	0.358	16/1558
CSP	0.9952	0.940	2/397
CelTOS	0.9884	1.530	3/182
SPECT2	0.9856	0.194	7/842
LSA1	0.9845	0.338	10/1162
LSA3	0.9989	0.358	5/478
UIS4	0.7190	0.451	1/108
P36	0.4100	0.071	2/379
UIS3	0.2100	0.038	2/229

**Table 2:** Relative surface accessibility of putative CD4+ and CD8+ T-cell epitopes.

Antigen	Strong-Binding CD4+ Epitope	Relative Surface Accessibility CD4+	Strong-Binding CD8+ Epitope	Relative Surface Accessibility CD8+
CELTOS	MNALRRLPVICS	Exposed	LPVICSFLVF	Exposed
CSP	KLAILSVSSFLF	Exposed	-	-
	LAILSVSSFLFV	Exposed	SSFLFVEALF *	Exposed
	ENWYSLKKNSRS	Exposed	-	-
LSA1	TNFKSLLRNLGV	Buried	-	-
	NFKSLLRNLGVS	Buried	-	-
	QTNFKSLLRNLG	Buried	-	-
	FKSLLRNLGVSE	Buried	KFIKSLFHIF *	Buried
	NFKSLLRNLGVS	Buried	-	-
	TNFKSLLRNLGV	Buried	-	-
	ISFYFILVNLLI	Buried	-	-
	SFYFILVNLLIF	Buried	-	-
LSA3	None	-	ASYVVGFFTF *	Buried
-	-	-	SYVVGFFTFS *	Buried
-	-	-	PFYSFVFDIF *	Buried
-	-	-	KVKNFVKKYK	Exposed
LSA3	-	-	KVDKNNKVPK *	Exposed
-	-	-	KTRKKAQRPK *	Buried
-	-	-	KVFAAPFISA *	Buried
-	-	-	KINKYFFLIK	Exposed
-	IRYNKSRSSNLI	Buried	-	-
-	AIRYNKSRSSNL	Buried	-	-
-	KFVQLLKSIDIK	Buried	-	-
-	RYNKSRSSNLIA	Buried	-	-
P36	FVQLLKSIDIKM	Buried	-	-
-	AIRYNKSRSSNL	Buried	KSKCETKIEK	Buried
-	EAIRYNKSRSSN	Buried	-	-
-	EEAIRYNKSRSS	Buried	-	-
-	IRYNKSRSSNLI	Buried	-	-
-	SKFVQLLKSIDI	Buried	-	-
-	MCYHFTMKRKKL	Exposed	-	-
-	HMCYHFTMKRKK	Exposed	-	-
-	NLFGLSSSKYIL	Buried	-	-
-	QNLFGLSSSKYI	Exposed	-	-
-	NININFVCSNVI	Buried	KYILFNNFLI	Buried
-	ININFVCSNVIQ	Buried	ILFNNFLILF *	Buried
P52	CYHFTMKRKKLF	Exposed	VYFIFLSFII *	Exposed
-	YHFTMKRKKLFV	Exposed	KVKHIMRINI	Buried
-	LFGLSSSKYILF	Buried	RTRTFWQNLF	Exposed
-	GTMIIYTKNINS	Buried	KLSRNHSFSS	Buried
-	MIIYTKNINSLM	Buried	NPSNCFHDVY	Buried
-	TMIIYTKNINSL	Buried	-	-
-	VGTMIIYTKNIN	Buried	-	-
-	FGLSSSKYILFN	Buried	-	-
-	-	-	VKYFNKPIQF	Exposed
-	-	-	YKYIQNIILF	Buried
-	-	-	YFMPKNDLNF	Buried
-	-	-	KYIQNIILFL	Buried
-	-	-	NYMPQNYYHI	Buried
SAP1	None	-	RIFFSFFSYF	Buried
-	-	-	RFKLTCNFKF	Buried
-	-	-	KLKNFFLNYK	Buried
-	-	-	KMTKNYNINA	Exposed
-	-	-	YTRAVWLLKK	Buried
-	-	-	MPKNDLNFIF	Buried
-	-	-	MPQNYYHINY	Buried
-	KLRILKKHYYVV *	Exposed	LYFIGIGYNL	Buried
-	LRILKKHYYVVF *	Exposed	IYVLCVDTTI	Buried
SPECT2	MKLRILKKHYYV *	Exposed	KRSKKTFLVK	Buried
-	MKLRILKKHYYV *	Exposed	KVVMFGFSLK	Buried
-	KLRILKKHYYVV *	Exposed	RSKKTFLVKS	Buried
-	LRILKKHYYVVF *	Exposed	KKIKHSFNLA	Exposed
-	-	-	YIPWDKTTAY	Buried
-	-	-	-	-
-	-	-	-	-
-	KYHLQKVYANYL *	Buried	-	-
-	YHLQKVYANYLS *	Buried	-	-
-	MEYMQKFVQGLQ *	Buried	-	-
-	NMEYMQKFVQGL *	Buried	-	-
UIS3	NNMEYMQKFVQG *	Buried	None	-
-	VNNMEYMQKFVQ *	Buried	-	-
-	LIYYSLIASGAI *	Exposed	-	-
-	IYYSLIASGAIA *	Exposed	-	-
	KQIIISTAAVAL *	Exposed	-	-
	QIIISTAAVALA *	Exposed	-	-
UIS4	RTLEKLLRKKQI *	Exposed	None	-
	DRTLEKLLRKKQ	Exposed	-	-
	LEKLLRKKQII *	Exposed	-	-

* Has one or more mutations relative to 3D7 epitope sequence. Underlined: mutations are predicted to influence peptide function.

## Data Availability

Whole-genome *Plasmodium falciparum* sequences originated from the MalariaGEN (www.malariagen.net/data/pf3k-5 (accessed on 9 March 2019) database.

## Supplementary Materials

The following supporting information can be downloaded at: https://www.mdpi.com/article/10.3390/microorganisms10061090/s1. Figure S1: Linear sequences of epitope fragments. All individual epitopes have been concatenated into a single fragment and polymorphic residues are highlighted in yellow. Figure S2: Predicted secondary structure and surface accessibility of malaria vaccine candidates. Figure S3: Predicted 3D structure of malaria vaccine candidates. Predicted protein–ligand binding sites are shown in light purple. Table S1: list of antigens selected and their putative function. Table S2: Sample origins. Table S3: FST values of the malaria vaccine candidates by continent. Table S4: FST values of the malaria vaccine candidates by country. Table S5: haplotype distribution by country. Table S6: Putative, strong-binding CD4+ and CD8+ T-cell epitopes in malaria vaccine candidates.
