# Supplementary material for: An In Silico Analysis of Malaria Pre-Erythrocytic-Stage Antigens Interpreting Worldwide Genetic Data to Suggest Vaccine Candidate Variants and Epitopes
Source: Microorganisms. 2022 May 25;10(6):1090. doi: 10.3390/microorganisms10061090 (PMC9231253; doi:10.3390/microorganisms10061090)
Supplement: Supplementary file 1 [file microorganisms-10-01090-s001.zip › microorganisms-1619852-supplementary.pdf]

Analyses of major malaria pre-erythrocytic stage antigens using worldwide isolates identify vaccine candidate variants and epitopes.

Supplemental Information

Amed Ouattara[1*], Dwivedi Ankit[2], Matthew Adams[1], Amadou Niangaly[3], Matthew B. Laurens[1], Myaing M. Nyunt[4], Christopher V. Plowe[3], Abdoulaye Djimde[3], Shannon Takala-Harrison[1], Joana C. Silva[2]

Author affiliation

[1]Malaria Research Program, Center for Vaccine Development and Global Heath, University of Maryland School of Medicine, 685 West Baltimore St., Baltimore, MD 21201, USA

[2]Institute for Genome Sciences, University of Maryland School of Medicine, 670 West Baltimore St., Baltimore, MD, 21201, USA

[3]Malaria Research and Training Center, University of Sciences, Techniques and Technologies of Bamako, P.O. Box: 1805, Bamako, Mali

[4]Duke Global Health Institute, Duke University, NC 27708

*Corresponding author

Email addresses:

AO: amouattara@som.umaryland.edu; AD: ADwivedi@som.umaryland.edu; MA: madams@som.umaryland.edu; AN: niangaly@icermali.org; MBL:

mlaurens@som.umaryland.edu; MM: myaing.nyunt@duke.edu; CVP: chris.plowe@duke.edu; AD: adjimde@icermali.org; STH: stakala@som.umaryland.edu; JCS: jcsilva@som.umaryland.edu

A. Figures

Supplemental Figure S1. Linear sequences of epitope fragments. All individual epitopes have been concatenated into a single fragment and polymorphic residues are highlighted in yellow.

- **P36**

EYIDKTNDVDIIATFNLICIKCKEPLLDKPMTNDKSYHLERIRREARKEGYITFTGFNF

- **UIS3**

LKKGIEDDLYYNKYLEYNNIKYRHVNMEYMVQGLQENNMY

- **UIS4**

VNDSEELD DKDKDE

- **CELTOS**

FRGNHSEQLESQVLPTVKKHAKSENLVAEPGAKVSDE

- **CSP**

ALYQCYG S SSTLNERSNKHKLKQPADGNPDNDPNEWSPCTCGNGIQVRIKPGSANKPKDEKKICKMEVN

- **SAP1**

RHVMFLRDDMNFEMNEQGKTHNLDIQSYMPYDQQKGENQEPSYLNKKEDVKIPKTKSCED NQYLIISELVNYHYCFYITDLCKKLSSVKYFNKPISILGNEQIQNGYQKLTSNHQMNQKILCDK NKKCDIPQKCDNTQDDENSEQNQNNSNGKIQHIDNSDHHIDPHLNEPSSNDVPKIQKHYRS

SIINVQKDISTQPTDDNNTAYLNRVLSHDILNEIQEIHDPDILISNLVQELKQHDIRTYYNDNAI TAPFNIQQTQTDNKDEYDKKNNVKNKHYNKAGNKFSSVQIDTQMDNPDKNDIYNSIINKSQ DGTCNTFLQNSLWKHFQILSIYKKEKCDQAKPYSNTDVLCRWTPSYICCNALNNYHYFTSTL CPQQTFKFTYDYKNK

- **LSA1**

VNVSRIKREDLEDLEQEEEEDLEEGEEDLEQQEEEEDLEEKEKQGQHSDEREHQSDEREHQSS LQDNRGNVEGRRKKIKPEQKEDDILETV NISDVDDEFDNQDNDNDKSLYDYI

- **LSA3**

SDKLEGQLVKSTVLDKVENLLTMSIIVIGETTEVIKDDNAKLKELKLSEDSKEIIDIEEADILKEL VLKGGRLVDKKEVDKEVSKASKPFISTFSLIAEKK

- **P52**

VILNNEFSTTAKTHNCPSRAPTPNFYSNMDIDFSCLCYGDKQDKVNIPSKRDLHAYNDVLGI NCFKKVIIPDPSCCKREFVGTMIINMDYDHDRTRIRTRTFWQNLL

- **SPECT2**

RSNCNSENDDMLASFSKHPLGEAILMLEGIANDLSTLQPVNGWIRKENACTLHVTCVIGLPP YPIGLPPYIPKTTAYKNAVELPAVFTGLDKESPSDVYNKSNLWMKFIINVSFSAGGSVDMNEQ LIVIGGNDSDIIYSPHDTDTKNSAPPQDTALKGYNIEVEAGSNSCTSKQSSNKYDTSYLY MECGDQPESTSTYPNYSILLGFGISSSGVYSTPCIPGMKSCSLNMSYIYVLALDGYSDGELVGTC LDGLTGFKVEFHEKCAKSLKACGSAK

Supplemental Figure S2. Predicted secondary structure and surface accessibility of malaria vaccine candidates.

Relative Surface Accessibility: Red is exposed and blue is buried, thresholded at 25%.
Secondary Structure: Helix, Strand, Coil.
Disorder: Thickness of line equals probability of disordered residue.

CSP

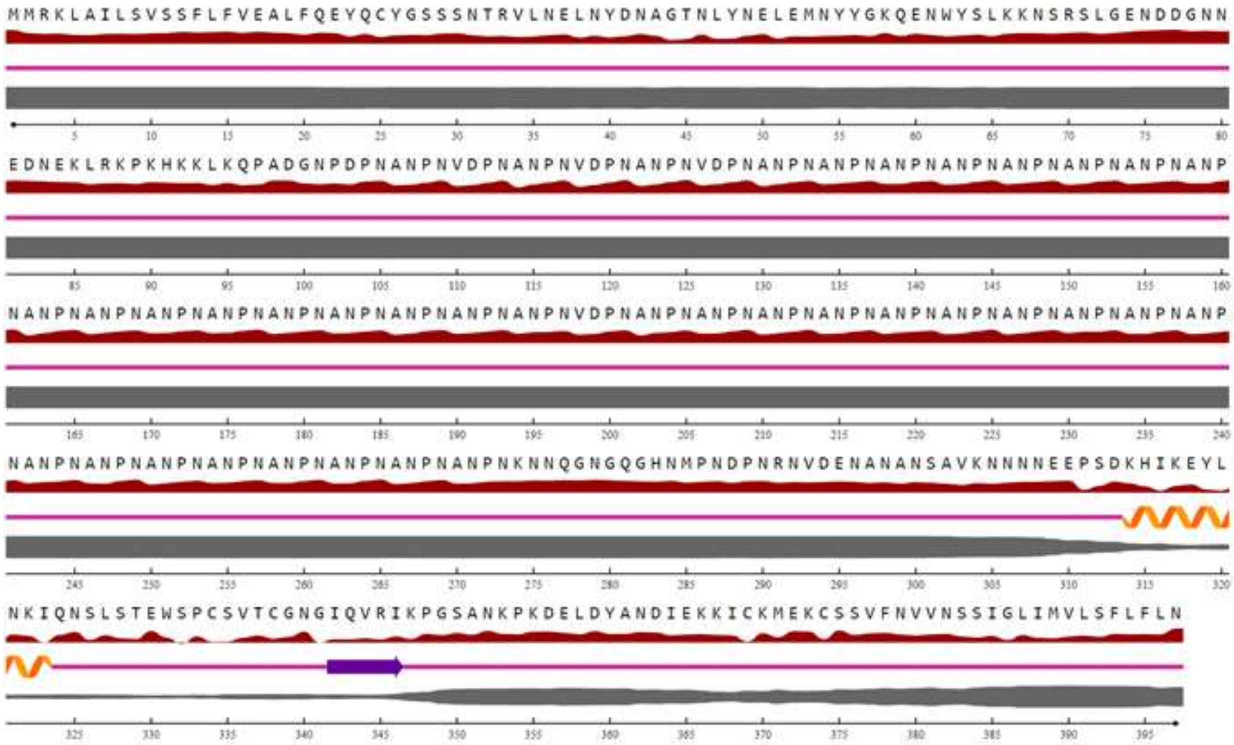

LSA1

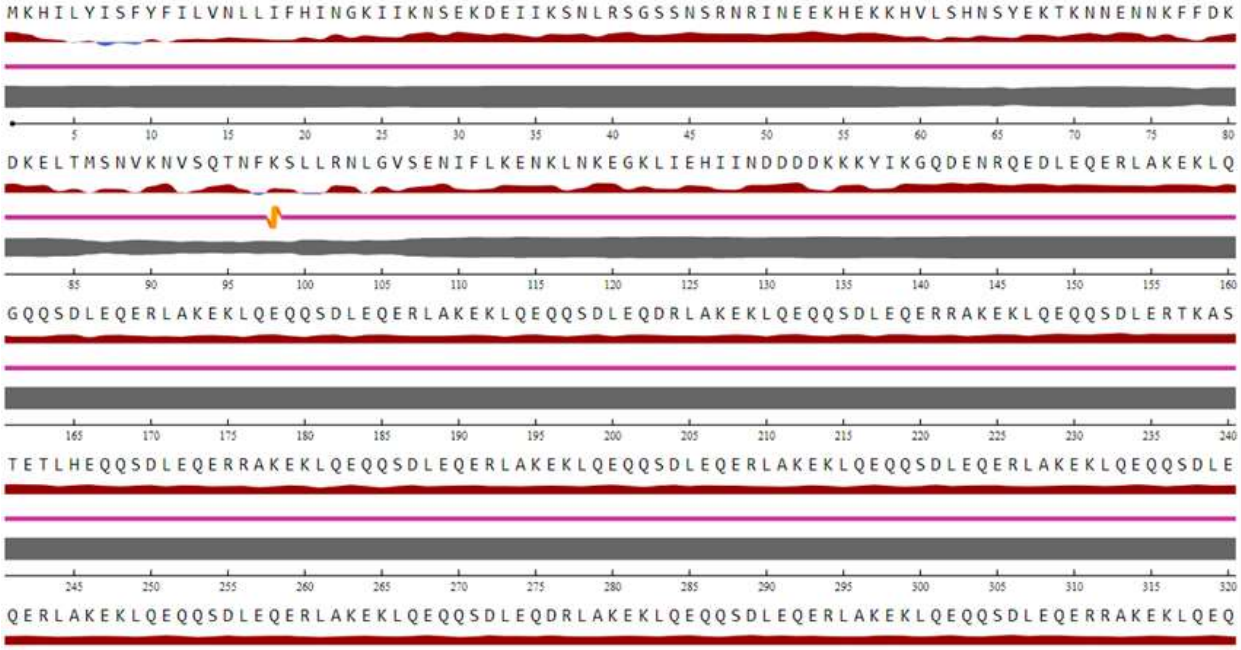

MKHILYISFYFILVNLLIFHINGKIIKNSEKDEIIKSNLRSGSSNSRNRINEEKHEKKHVLSHNSYEKTKNNENNKFFDK
5 10 15 20 25 30 35 40 45 50 55 60 65 70 75 80
DKELTMSNVKNVSQTNFKSLLRNLGVSENIFLKENKLNKEGKLIEHIINDDDDKKKYIKGQDENRQEDLEQERLAKEKLQ
85 90 95 100 105 110 115 120 125 130 135 140 145 150 155 160
GQQSDLEQERLAKEKLQEQQSDLEQERLAKEKLQEQQSDLEQDRLAKEKLQEQQSDLEQERRAKEKLQEQQSDLERTKAS
165 170 175 180 185 190 195 200 205 210 215 220 225 230 235 240
TETLHEQQSDLEQERRAKEKLQEQQSDLEQERLAKEKLQEQQSDLEQERLAKEKLQEQQSDLEQERLAKEKLQEQQSDLE
245 250 255 260 265 270 275 280 285 290 295 300 305 310 315 320
QERLAKEKLQEQQSDLEQERLAKEKLQEQQSDLEQDRLAKEKLQEQQSDLEQERLAKEKLQEQQSDLEQERRAKEKLQEQ

LSA 3

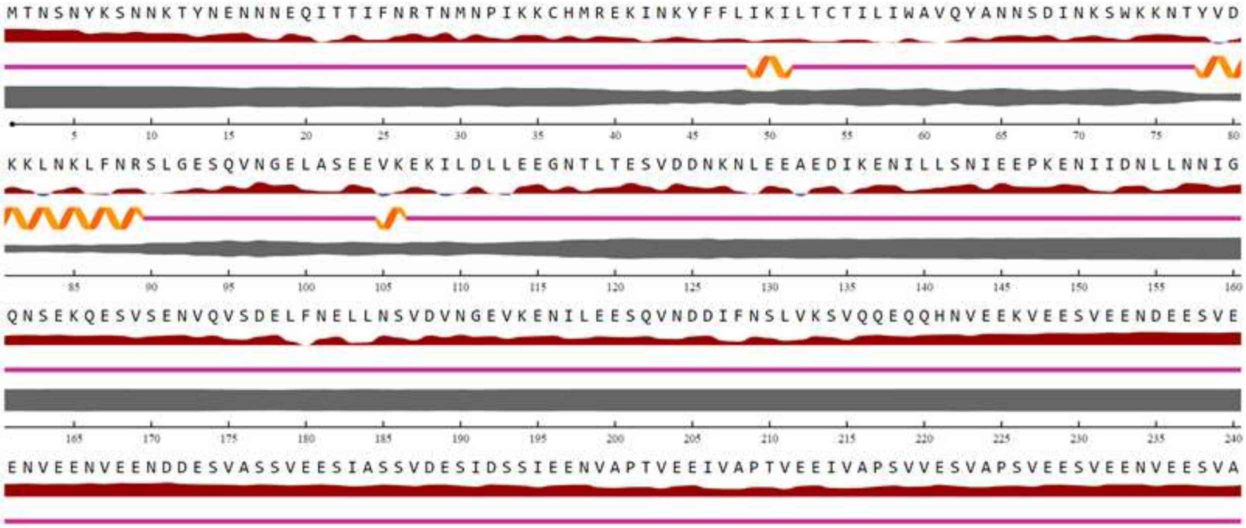

MTNSNYKSNNKTYNENNNEQITTIFNRTNMNPIKKCHMREKINKYFFLIKILTCTILIWAVQYANNSDINKSWKKNTYVD
5 10 15 20 25 30 35 40 45 50 55 60 65 70 75 80
KKLNKLFNRSLGESQVNGELASEEVKEKILDLLEEGNTLTESVDDNKNLEEAEDIKENILLSNIEEPKENIIDNLLNNIG
85 90 95 100 105 110 115 120 125 130 135 140 145 150 155 160
QNSEKQESVSENVQVSDELFNELLNSVDVNGEVKENILEESQVNDDIFNSLVKSVQQEQQHNVEEKVEESVEENDEESVE
165 170 175 180 185 190 195 200 205 210 215 220 225 230 235 240
ENVEENVEENDDESVASSVEESIASSVDESIDSSIEENVAPTVEEIVAPTVEEIVAPSVVESVAPSVEESVEENVEESVA

P52

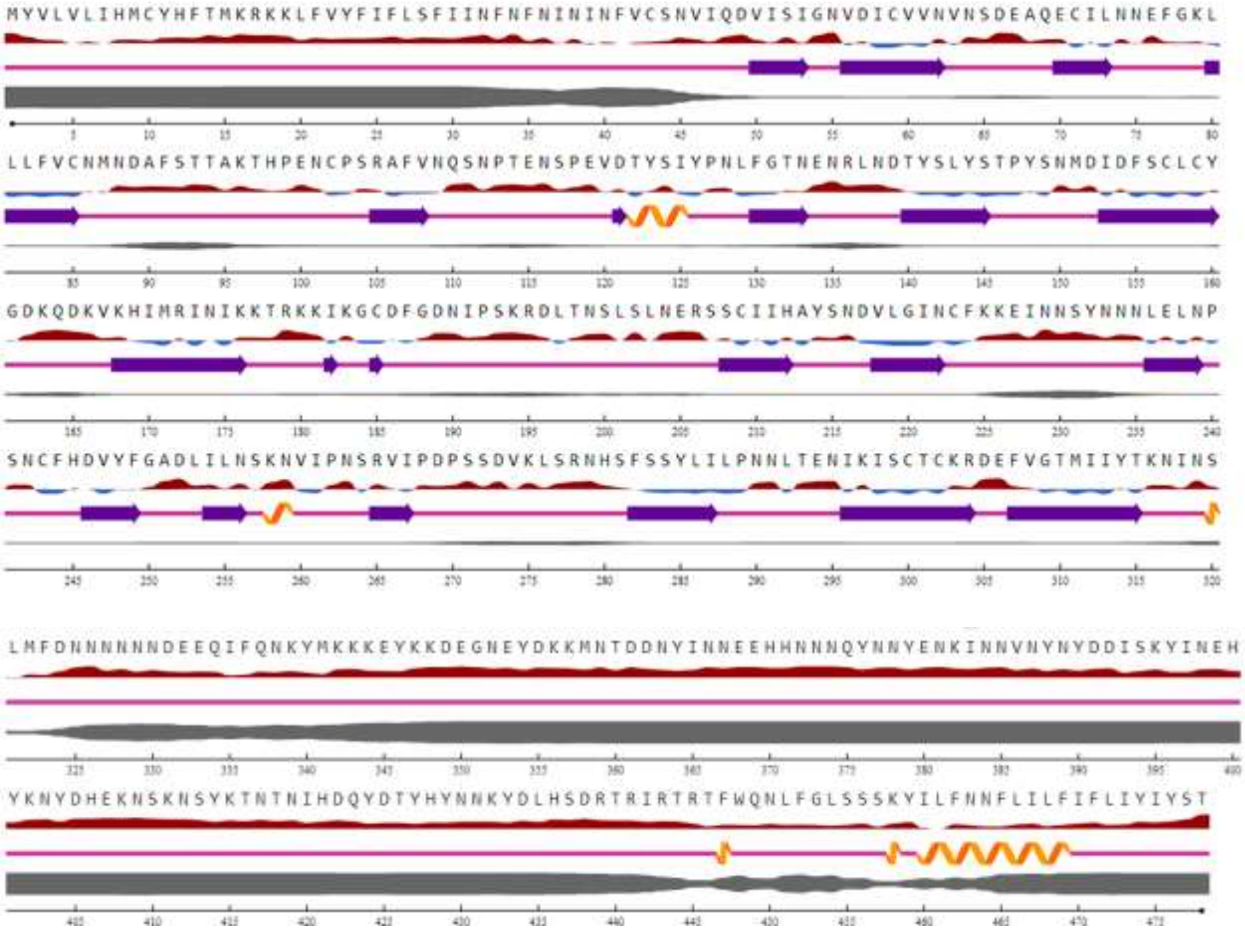
MYVLVLIHMCYHFTMKRKKLFVYFIFLSFIINFNFNININFVCSNVIQDVISIGNVDICVVNVNSDEAQECILNNEFGKL
LLFVCNMNDAFSTTAKTHPENCPSRAFVNQSNPTENSPEVDTYSIYPNLFGTNENRLNDTYSLYSTPYSNMDIDFSCLCY
GDKQDKVKHIMRINIKKTRKKIKGCDFGDNIPSKRDLTNSLSLNERSSCIIHAYSNDVLGINCFKKEINNSYNNNLELNP
SNCFHDVYFGADLILNSKNVIPNSRVIPDPSSDVKLSRNHSFSSYLILPNNLTENIKISCTCKRDEFVGTMIIYTKNINS
LMFDNNNNNNDEEQIFQNKYMKKKEYKKDEGNEYDKKMNTDDNYINNEEHHNNNQYNNYENKINNVNYNYDDISKYINEH
YKNYDHEKNSKNSYKTNTNIHDQYDTYHYNNKYDLHSDRTRIRTRTFWQNLFGLSSSKYILFNNFLILFIFLIYIYST

SPECT2

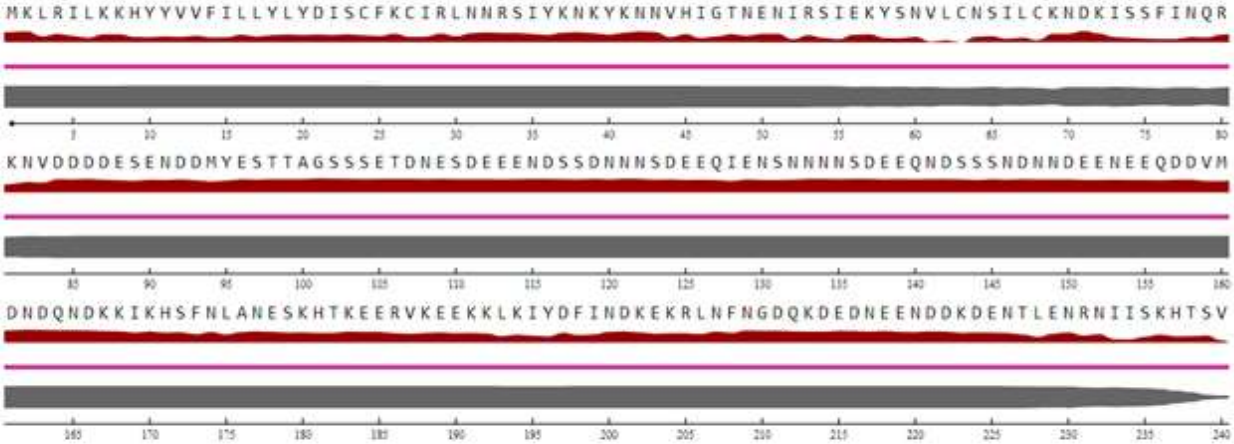
MKLRILKKHYYVVFILLYLYDISCFKCIRLNNRSIYKNKYKNNVHIGTNENIRSIEKYSNVLCNSILCKNDKISSFINQR
KNVDDDDESENDDMYESTTAGSSSETDNESDEEENDSSDNNNSDEEQIENSNNNNSDEEQNDSSSNDNNDEENEEQDDVM
DNDQNDKKIKHSFNLANESKHTKEERVKEEKKLKIYDFINDKEKRLNFNGDQKDEDNEENDDKDENTLENRNIISKHTSV

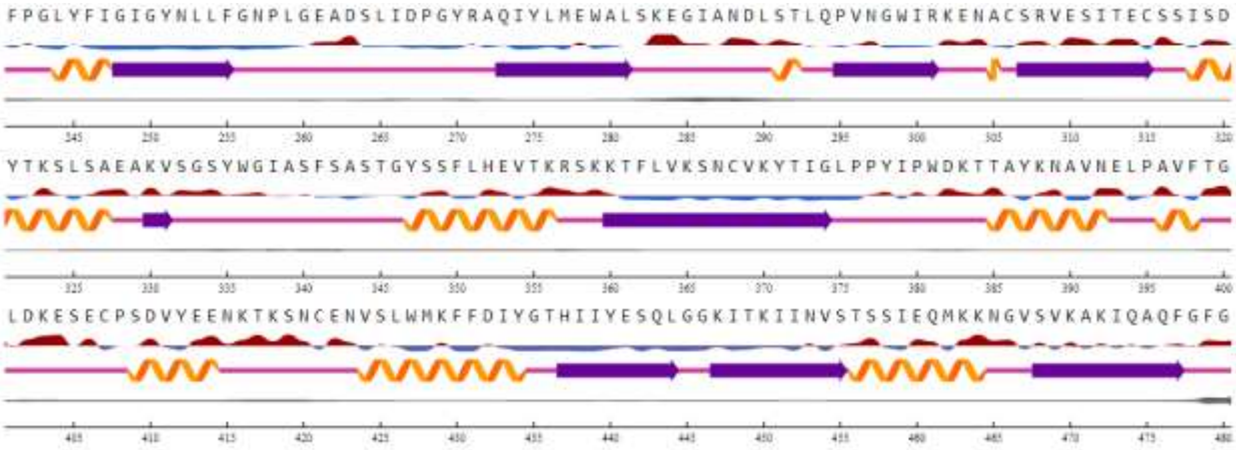
FPGLYFIGIGYNLLFGNPLGEADSLIDPGYRAQIYLMEWALSKEGIANDLSTLQPVNGWIRKENACSRVESITECSSISD
YTKSLSAEAKVSGSYWGIASFSASTGYSSFLHEVTKRSKKTFLVKSNCVKYTIGLPPYIPWDKTTAYKNAVNELPAVFTG
LDKESECPSDVYEENKTKSNCENVSLWMKFFDIYGTHIIYESQLGGKITKIINVSTSSIEQMKKNGVSVKAKIQAQFGFG

UIS3

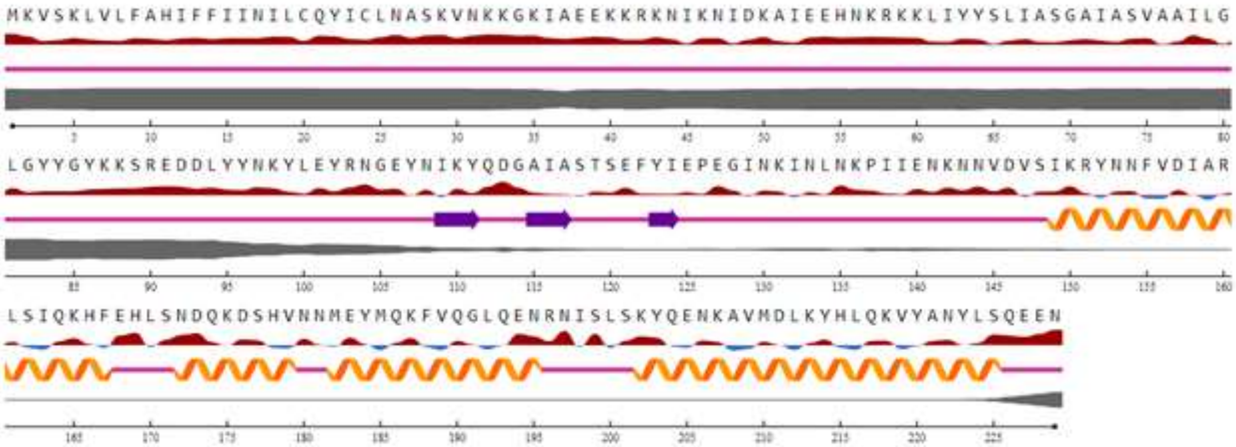
MKVSKLVLFAHIFFIINILCQYICLNASKVNKKGKIAEEKKRKNIKNIDKAIEEHNKRKKLIYYSLIASGAIASVAAILG
LGYYGYKKSREDDLYYNKYLEYRNGEYNIKYQDGAIASTSEFYIEPEGINKINLNKPIIENKNNVDVSIKRYNNFVDIAR
LSIQKHFEHLSNDQKDSHVNNMEYMQKFVQGLQENRNISLSKYQENKAVMDLKYHLQKVVYANYLSQEEN

UIS4

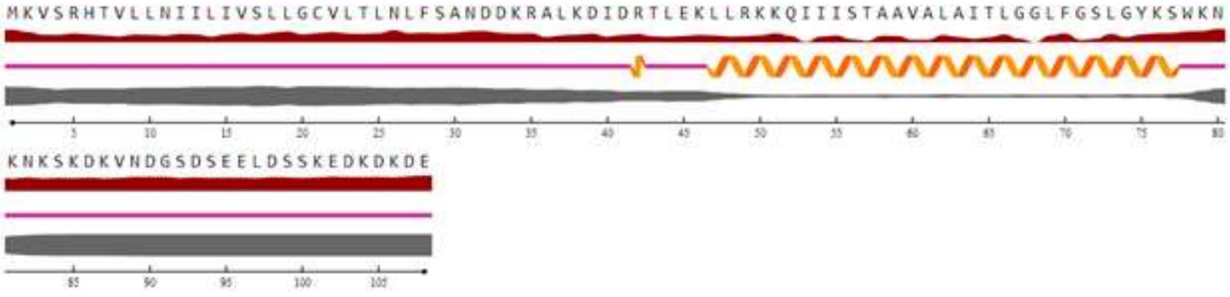
MKVSRHTVLLNIILIVSLLGCVLTLNLFSANDDKRALKDIDRTLEKLLRKKQIIISTAAVALAITLGGLFGSLGYKSWKN
KNKSKDKVNDGSDSEELDSSKEDKDKDE

P36

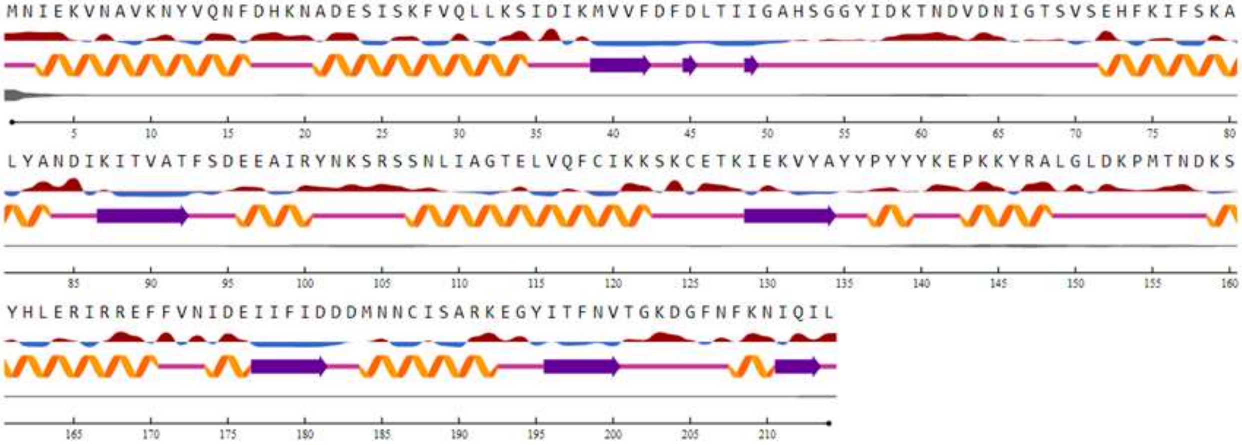
MNIEKVNAVKNYVQNFDHKNADESISKFVQLLKSIDIKMVVFDFDLTIIGAHSGGYIDKTNDVDNIGTSVSEHFKIFSKA
LYANDIKITVATFSDEEAIRYNKSRSSNLIAGTELVQFCIKKSKCETKIEKVYAYYPYYYKEPKKYRALGLDKPMTNDKS
YHLERIRREFFVNIDEIIFIDDDMNNCISARKEGYITFNVTGKDGFNFKNIQIL

CELTOS

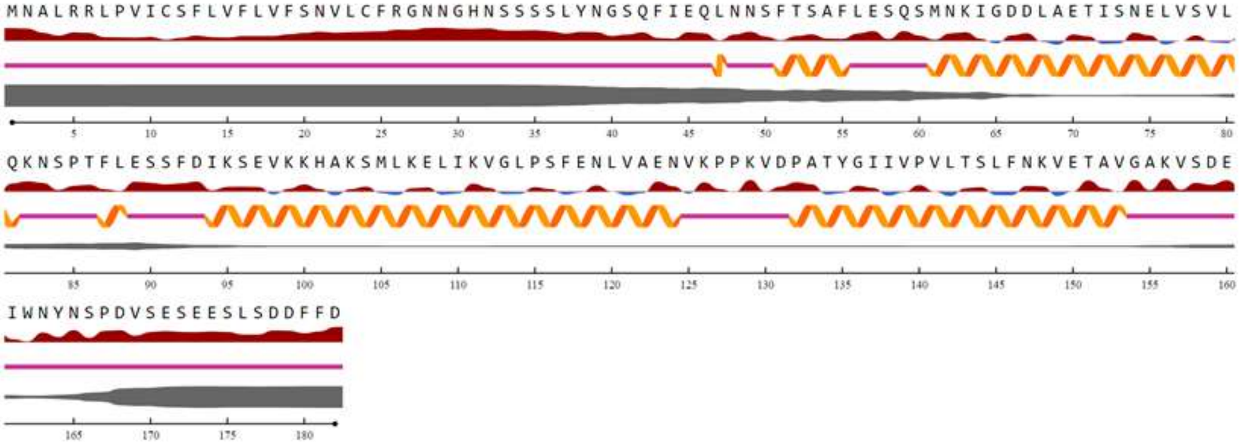
MNALRRLPVICSFLVFLVFSNVLCFRGNNGHNSSSSLYNGSQFIEQLNNSFTSAFLESQSMNKIGDDLAETISNELVSVL
QKNSPTFLESSFDIKSEVKKHAKSMLKELIKVGLPSFENLVAENVKPPKVDPATYGIIVPVLTSLFNKVETAVGAKVSDE
IWNYNSPDVSESEESLSDDFFD

SAP 1

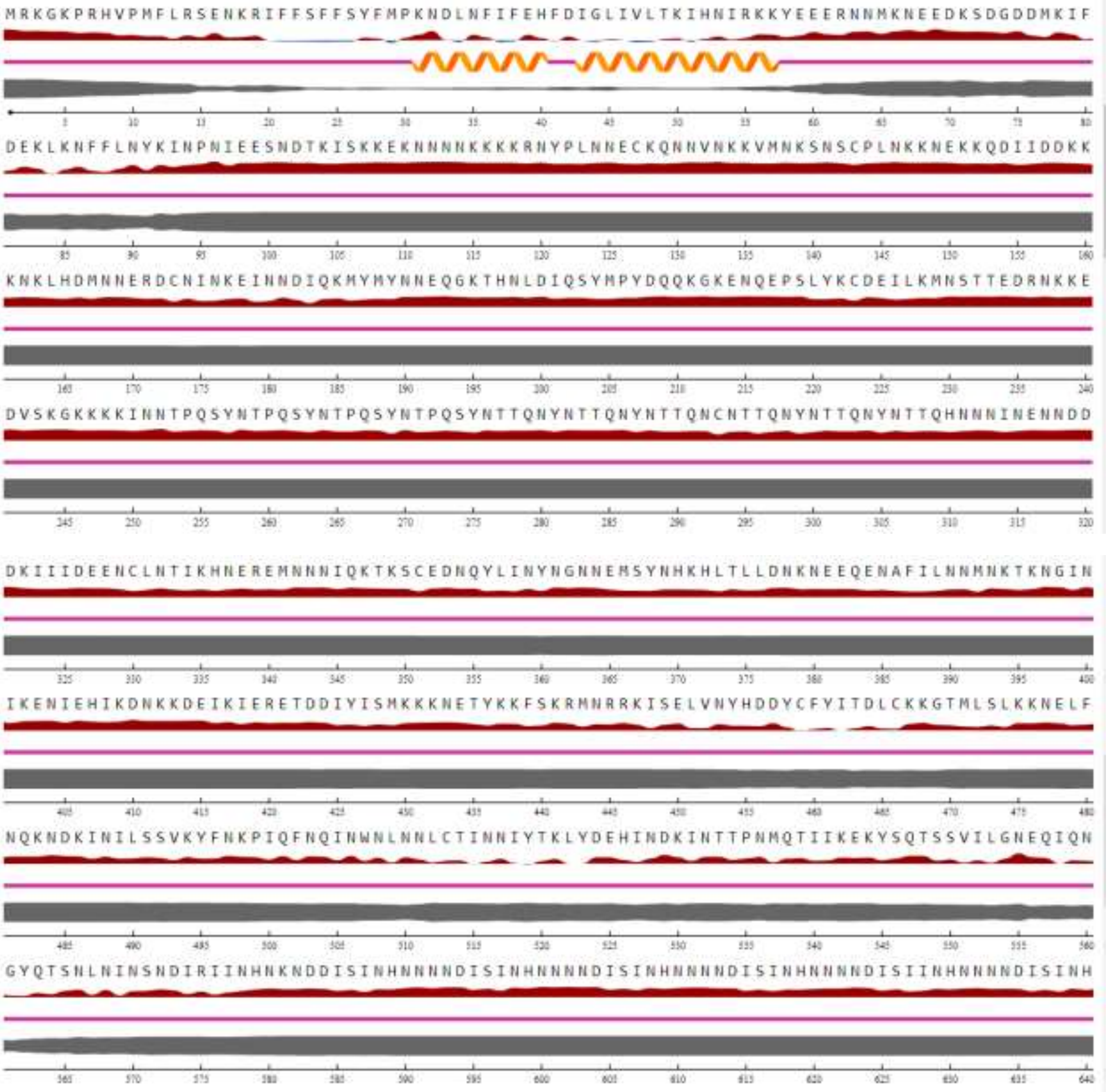
MRKGKPRHVPMFLRSENKRIFFSFFSYFMPKNDLNFIFEHFDIGLIVLTKIHNIRKKYEEERNNMKNEEDKSDGDDMKIF
DEKLKNFFLNYKINPNIEESNDTKISKKEKNNNNKKKKRNYPLNNECKQNNVNKKVMNKSNSCPLNKKNEKKQDIIDDKK
KNKLHDMNNERDCNINKEINNDIQKMYMYNNEQGKTHNLDIQSYMPYDQQKGKENQEPSLYKCDEILKMNSTTEDRNKKE
DVSKGKKKKINNTPQSYNTPQSYNTPQSYNTPQSYNTTQNYNTTQNYNTTQNCNTTQNYNTTQNYNTTQHNNNINENNDD
DKIIIDEENCLNTIKHNEREMNNNIQKTKSCEDNQYLINYNGNNEMSYNHKHLTLLDNKNEEQENAFILNNMNKTKNGIN
IKENIEHIKDNKKDEIKIERETDDIYISMKKKNETYKKFSKRMNRRKISELVNYHDDYCFYITDLCKKGTMLSLKKNELF
NQKNDKINILSSVKYFNKPIQFNQINWNLNNLCTINNIYTKLYDEHINDKINTTPNMQTIIKEKYSQTSSVILGNEQIQN
GYQTSNLNINSNDIRIINHNKNDDISINHNNNNDISINHNNNNDISINHNNNNDISINHNNNNDISIINHNNNNDISINH

NNNNNNYYFMNNYMHNNINNNYYCYYMNNTNHVNNYYNNIYIQNNHDQNNAPILQPINNHLAHINDLCYIHSEKNEYTKI

645 650 655 660 665 670 675 680 685 690 695 700 705 710 715 720

SKNHQMNNINPQQSNGKNQNDISNNINKNDEYYNLNEQKILCDKNKSYIKCDIPQKCDNTQDDENSEQNQNYITNPSNGH

725 730 735 740 745 750 755 760 765 770 775 780 785 790 795 800

YKIKEQMNYVQHIPDYEHDNTTNEMINTQNYTNINLDPYIMNNQNDNNVYLNQNYFDTEKKNKEEDINITEANTHYNNVH

805 810 815 820 825 830 835 840 845 850 855 860 865 870 875 880

IYQNNSNHMNKMINLNNNTNSKSTQDYDLYHNNMENFNNTNYNIMKEKIHINDDTSSVINNSSINSQNNLCNNKKTNNDY

885 890 895 900 905 910 915 920 925 930 935 940 945 950 955 960

QNRNIKNDNSIPDSSINMNELKNNIQMNDYYASNIYNNNNNNNNNNFIISNNIISNYNNYMNESNVYPQINSNNYIPYID

965 970 975 980 985 990 995 1000 1005 1010 1015 1020 1025 1030 1035 1040

PHLNNEPYKHTINNQLNKNCINNNISMDDNVYDSNTISIHISVNDNEYNNSNFTNDMYNNNNNNNNNNNNCNNSSNSNSN

1045 1050 1055 1060 1065 1070 1075 1080 1085 1090 1095 1100 1105 1110 1115 1120

SNQVCLYMPNHELYNSNYNITQHMPSSNDVPKIQKHIANNIIMNGHKEEHIIEKKSKEETNKTNEQVYRSINQNNTLILK

1125 1130 1135 1140 1145 1150 1155 1160 1165 1170 1175 1180 1185 1190 1195 1200

ENEIDENDINTLNQNLNIKNDMNNMDNIINLKNLNNINNIYTPYQNNILKNNEIQFLNNNKEVITKHAYTHSSNEININV

1205 1210 1215 1220 1225 1230 1235 1240 1245 1250 1255 1260 1265 1270 1275 1280

YKNIDTQINIRKNENNNDIIINKEQKDISTNNEQNQTYNYITSSVKNEYSILDNSPTDKNYKKNLNLNISSSYNTNDHIN

1285 1290 1295 1300 1305 1310 1315 1320 1325 1330 1335 1340 1345 1350 1355 1360

NYHDDIDKTNKDNKYEEEKHKDINIYINNINSNGNKNHNFINSYFDLNENEKKKKINIFYHDNKSLKNISNEELTNTNPY

1365 1370 1375 1380 1385 1390 1395 1400 1405 1410 1415 1420 1425 1430 1435 1440

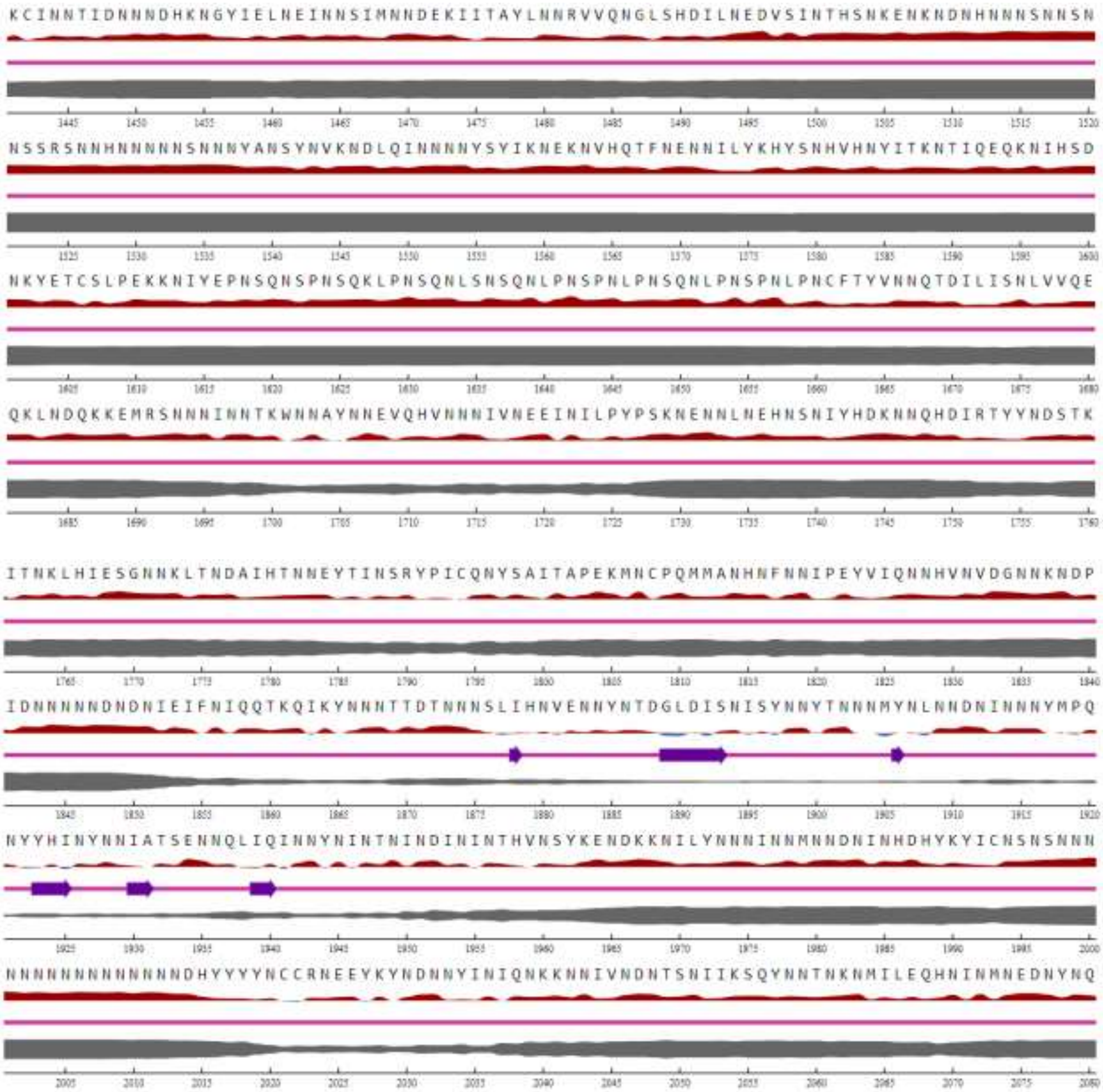
KCINNTIDNNNDHKNGYIELNEINNSIMNNDEKIITAYLNNRVVQNGLSHDILNEDVSINTHSNKENKNDNHNNNSNNSN
1445 1450 1455 1460 1465 1470 1475 1480 1485 1490 1495 1500 1505 1510 1515 1520
NSSRSNNHNNNNNSNNNYANSYNVKNDLQINNNNYSYIKNEKNVHQTFNENNILYKHYSNHVHNYITKNTIQEQKNIHSD
1525 1530 1535 1540 1545 1550 1555 1560 1565 1570 1575 1580 1585 1590 1595 1600
NKYETCSLPEKKNIYEPNSQNSPNSQKLPNSQNLSNSQNLPNSPNLPNSQNLPNSPNLPNCFTYVNNQTDILISNLVVQE
1605 1610 1615 1620 1625 1630 1635 1640 1645 1650 1655 1660 1665 1670 1675 1680
QKLNDQKKEMRSNNNINNTKWNNAYNNEVQHVNNNIVNEEINILPYPSKNENNLNEHNSNIYHDKNNQHDIRTYYNDSTK
1685 1690 1695 1700 1705 1710 1715 1720 1725 1730 1735 1740 1745 1750 1755 1760
ITNKLHIESGNNKLTNDAIHTNNEYTINSRYPICQNYSAITAPEKMNCPQMMANHNFNNIPEYVIQNNHVNVDGNNKNDP
1765 1770 1775 1780 1785 1790 1795 1800 1805 1810 1815 1820 1825 1830 1835 1840
IDNNNNNDNDNIEIFNIQQTKQIKYNNNTTDTNNNSLIHNVENNYNTDGLDISNISYNNYTNNNMYNLNNDNINNNYMPQ
1845 1850 1855 1860 1865 1870 1875 1880 1885 1890 1895 1900 1905 1910 1915 1920
NYYHINYNNIATSENNQLIQINNYNINTNINDININTHVNSYKENDKKNILYNNNINNMNNDNINHDHYKYICNSNSNNN
1925 1930 1935 1940 1945 1950 1955 1960 1965 1970 1975 1980 1985 1990 1995 2000
NNNNNNNNNNNNNDHYYYYNCCRNEEYKYNDNNYINIQNKKNNIVNDNTSNIIKSQYNNTNKNMILEQHNINMNEDNYNQ
2005 2010 2015 2020 2025 2030 2035 2040 2045 2050 2055 2060 2065 2070 2075 2080

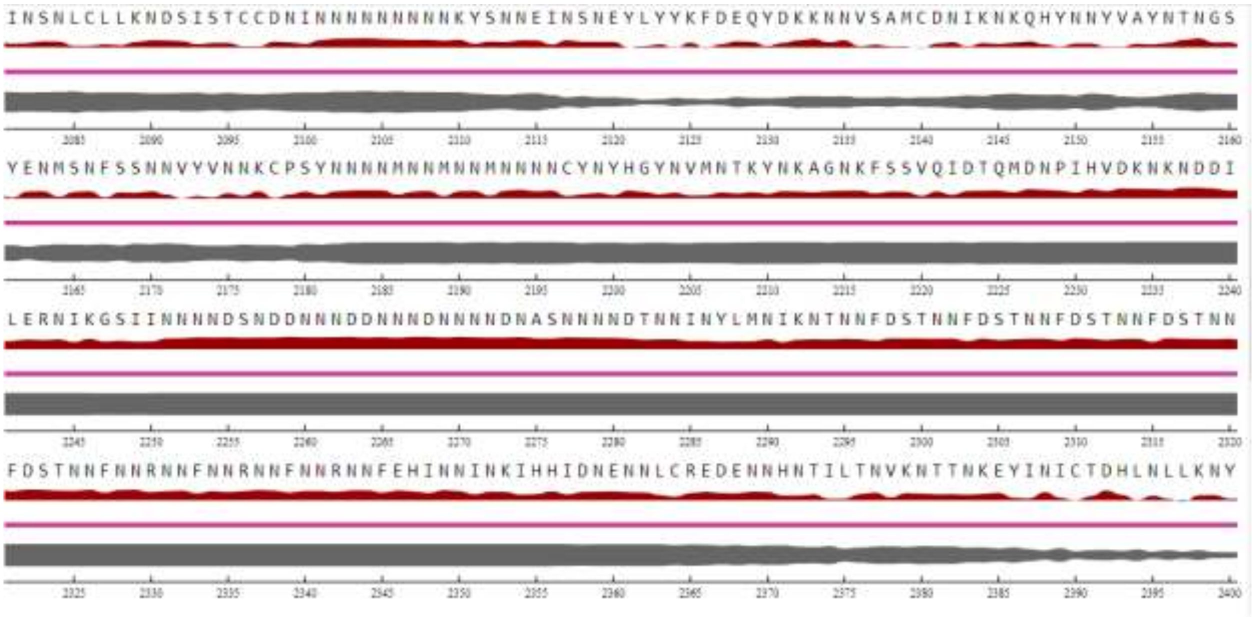

INSNLCLLKNDSISTCCDNINNNNNNNNNKYSNNEINSNEYLYYKFDEQYDKKNNVSAMCDNIKNKQHYNNYVAYNTNGS
YENMSNFSSNNVYYVNNKCPSYNNNNMNNMNNMNNNNCYNYHGYNVMNTKYNKAGNKFSSVQIDTQMDNPIHVDKNKNDDI
LERNIKGSIINNNNDSNDDNNNDDNNNDNNNNDNASNNNNDTNNINYLMNIKNTNNFDSTNNFDSTNNFDSTNNFDSTNN
FDSTNNFNNRNNFNNRNNFNNRNNFEHINNINKIHHIDNENNLCREDENNHNTILTNVKNTTNKEYINICTDHLNLLKNY

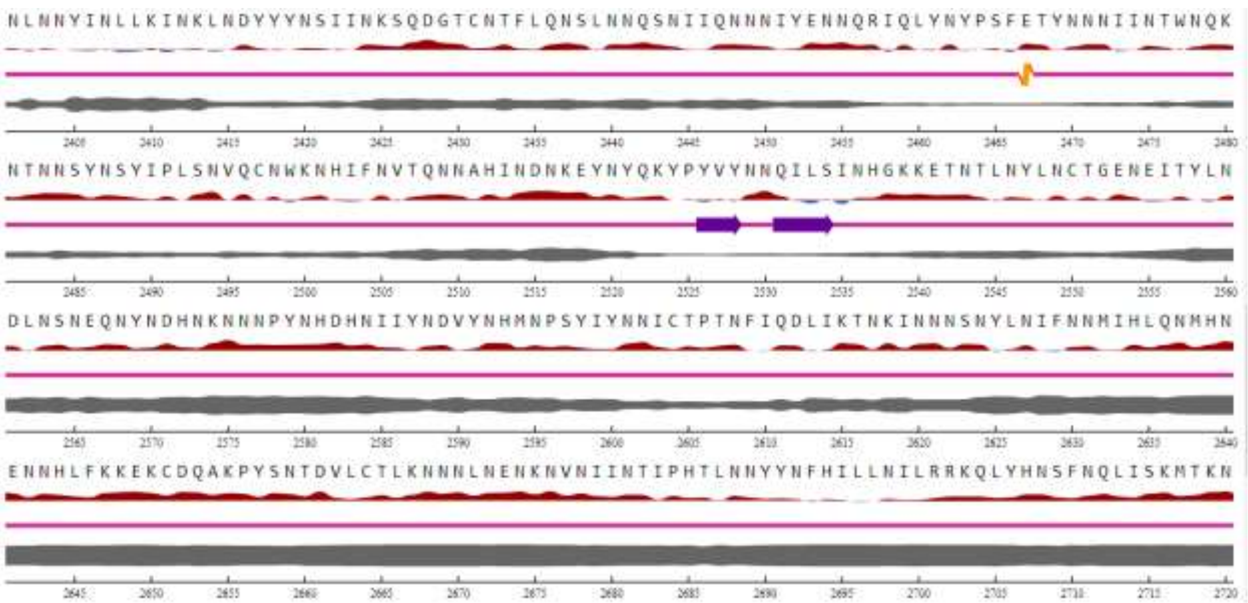

NLNNYINLLKINKLNDYYYNSIINKSQDGTCNTFLQNSLNNQSNIIQNNNIYENNQRIQLYNYPSFETYNNNIINTWNQK
NTNNSYNSYIPLSNVQCNWKNHIFNVTQNNAHINDNKEYNYQKYPYVVYNNQILSINHGKKETNTLNYLNCTGENEITYLN
DLNSNEQNYNDHNKNNNPYNHDHNIIYNDVYNHMNPSYIYNNICTPTNFIQDLIKTNKINNNSNYLNIFNNMIHLQNMHN
ENNHLFKKEKCDQAKPYSNTDVLCTLKNNNLNENKNVNIINTIPHTLNNYYNFHILLNILRRKQLYHNSFNQLISKMTKN

Supplemental Figure S3. Predicted 3D structure of malaria vaccine candidates. Hypothetical protein-ligand binding sites are shown in light purple.

CSP

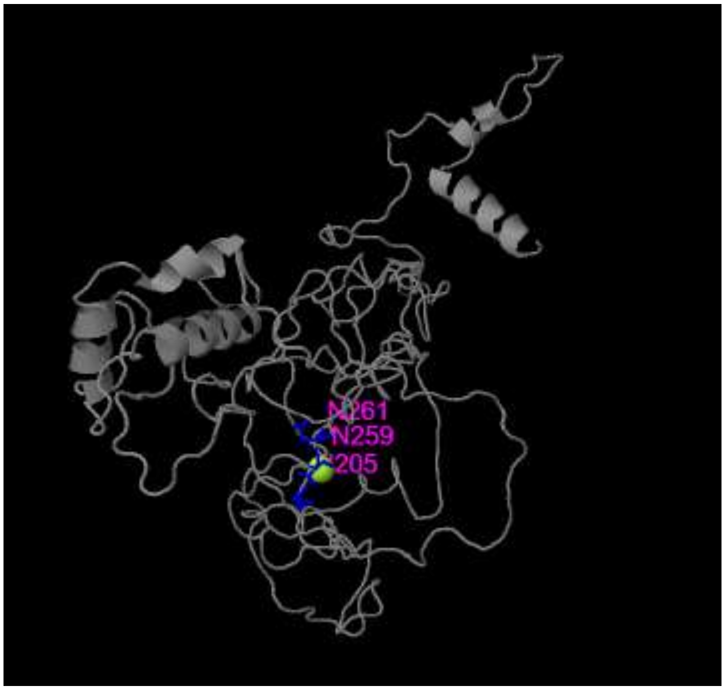

LSA1

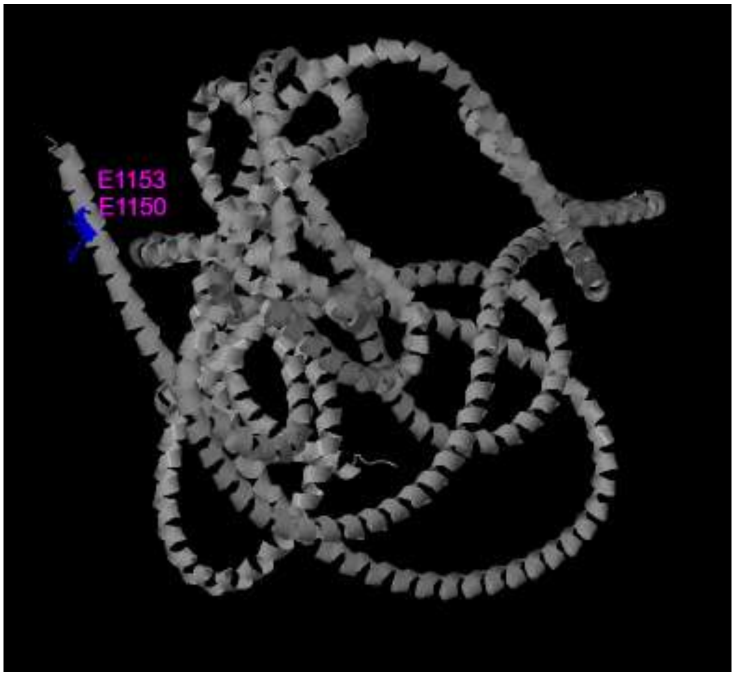

LSA3

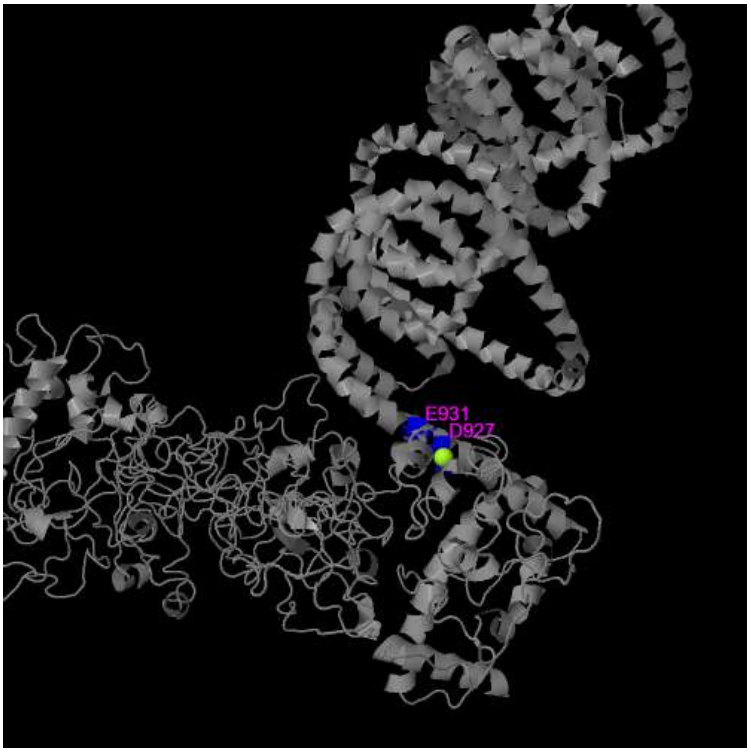

SPECT2

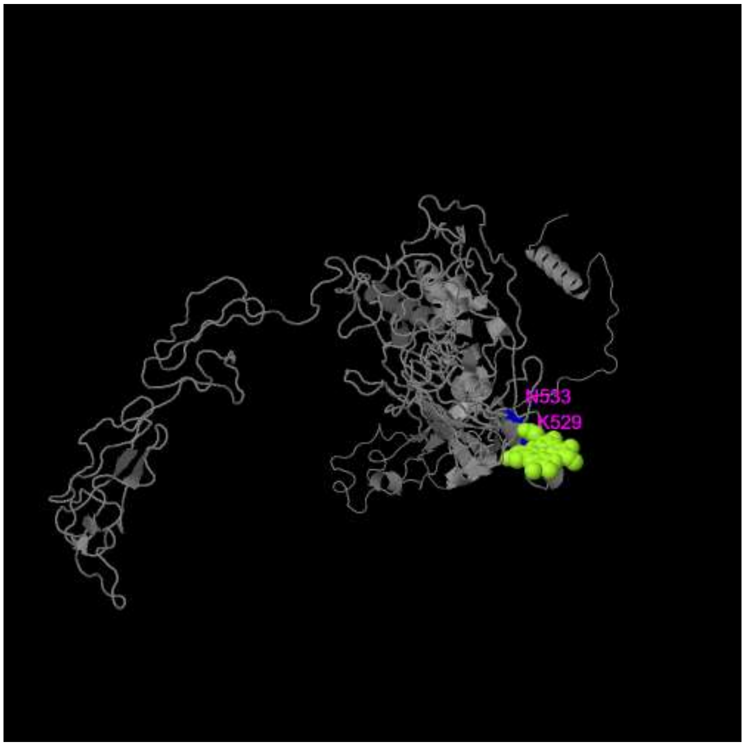

P52

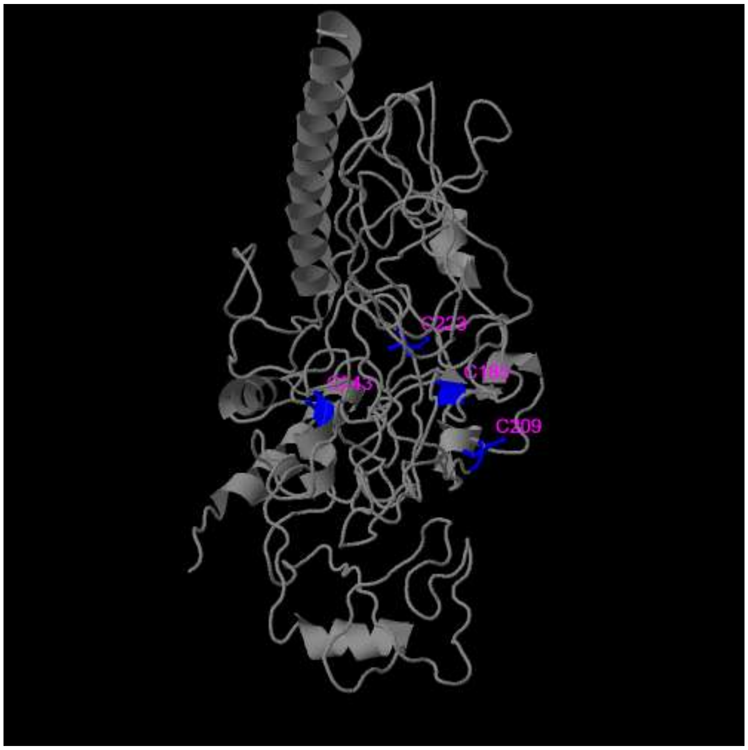

UIS3

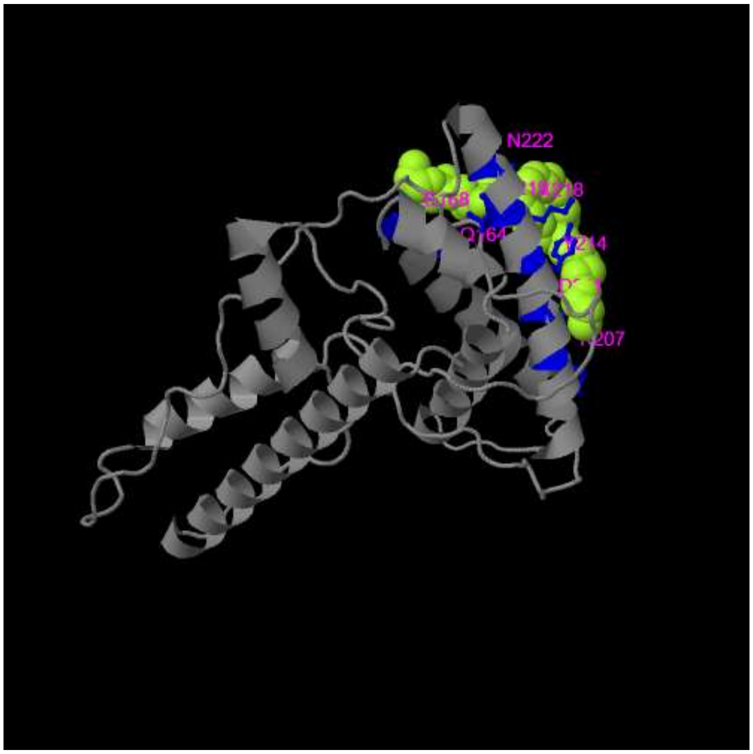

UIS4

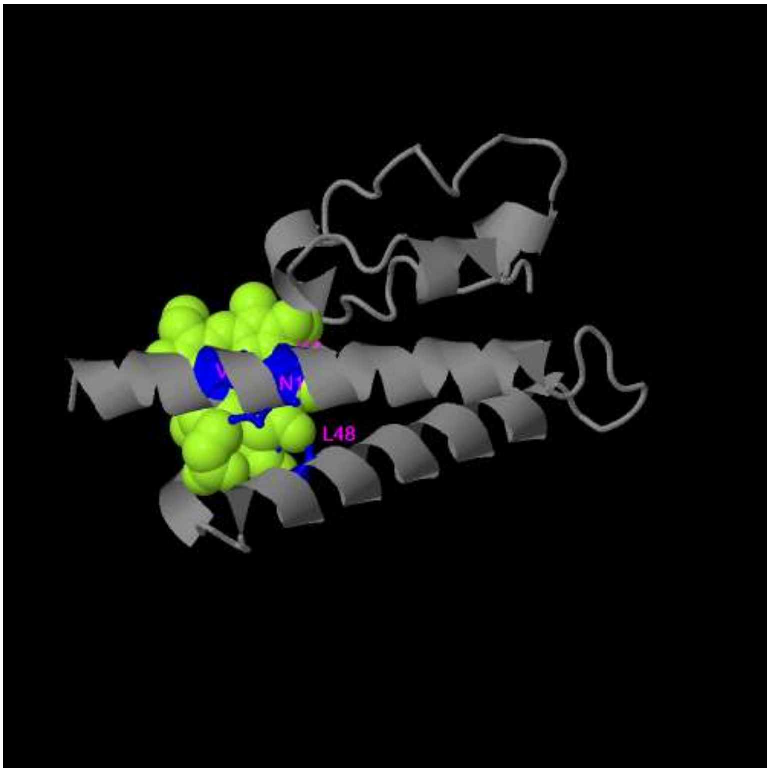

P36

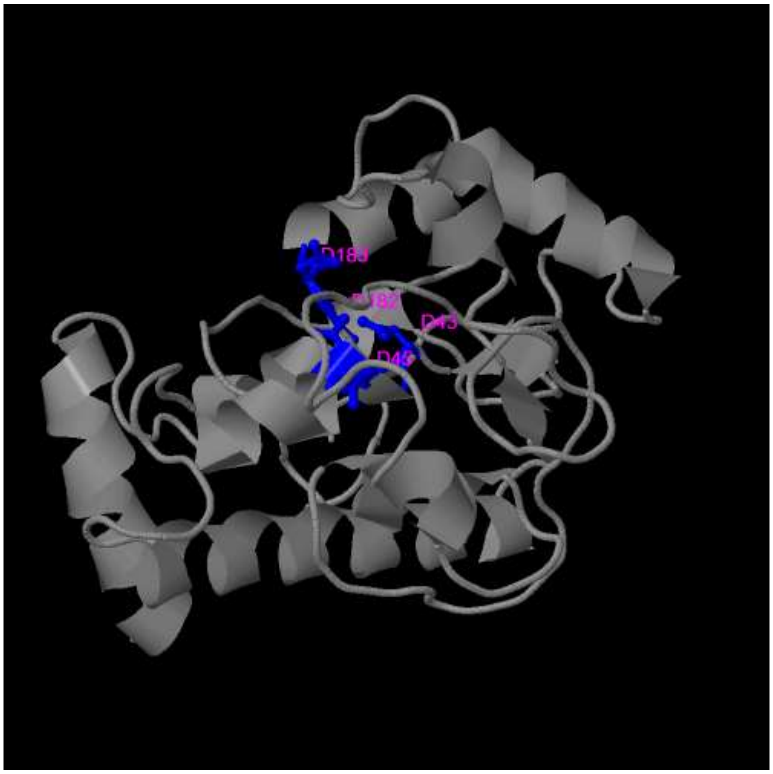

CELTOS

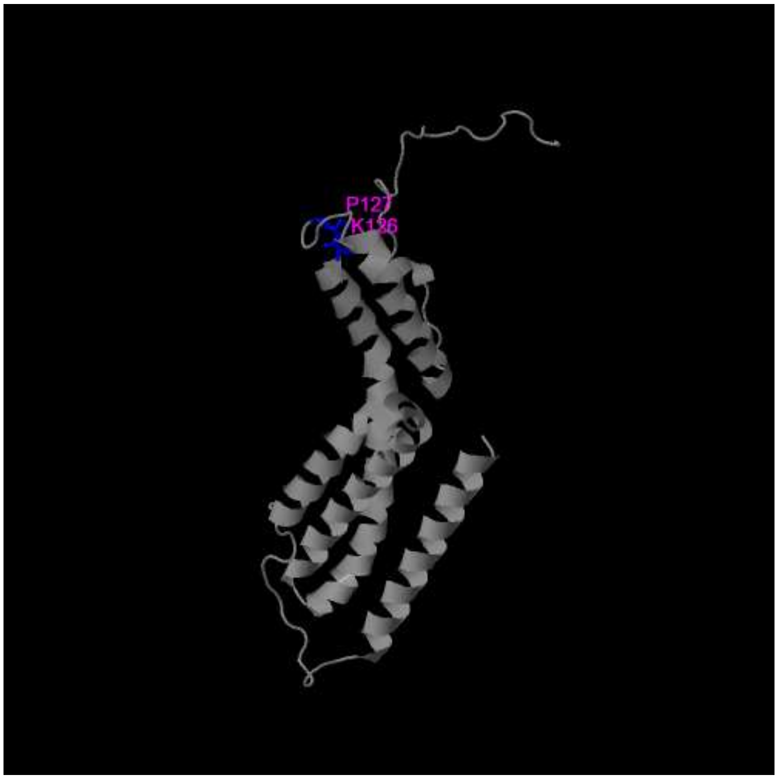

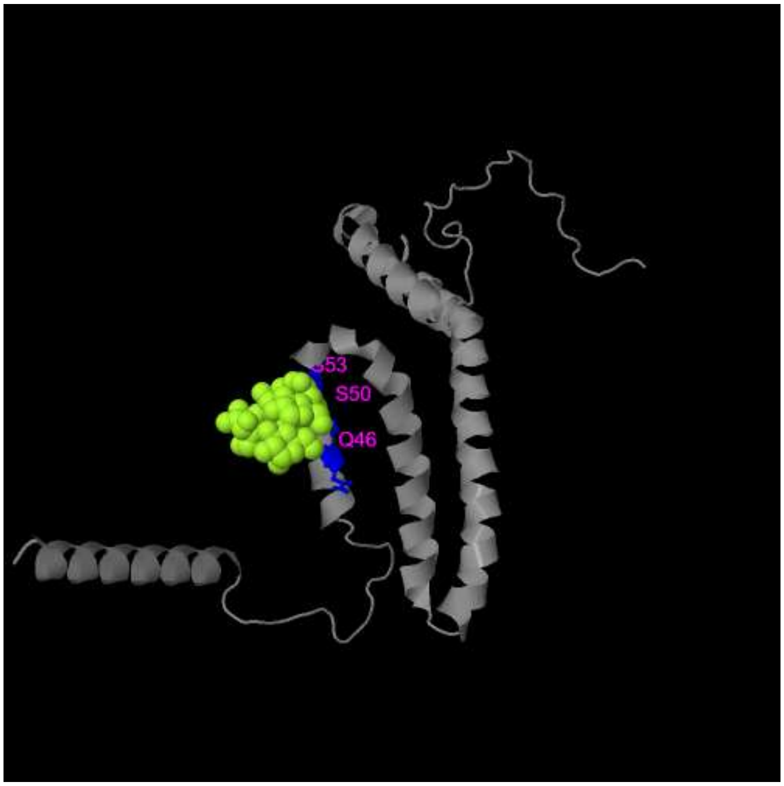

Three fragments of SAP1

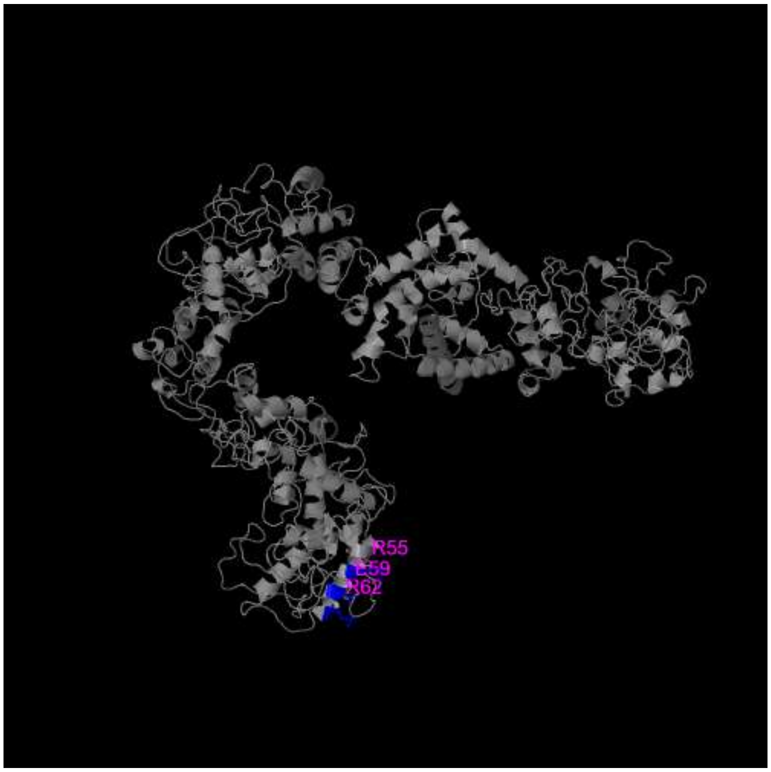

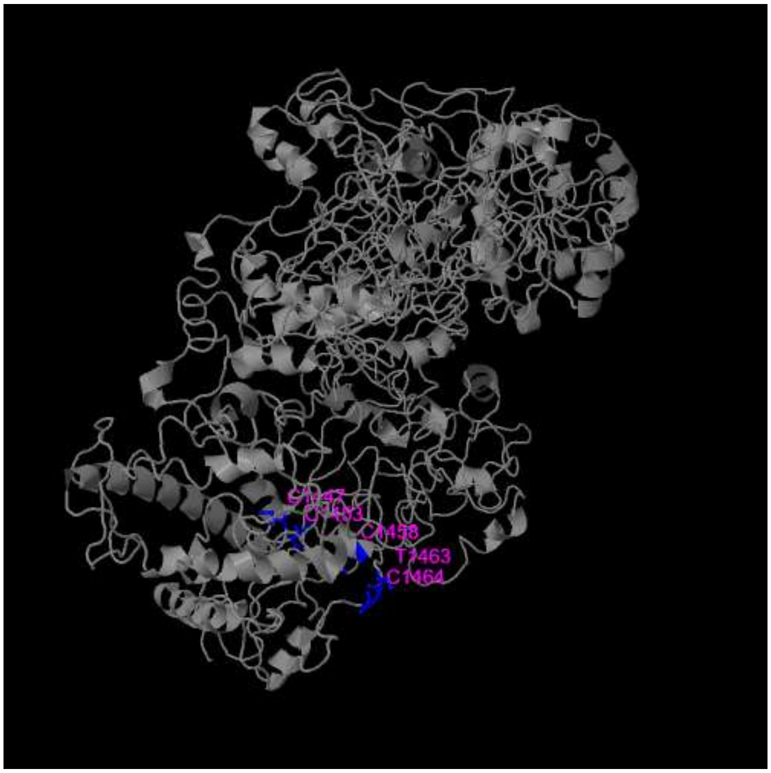
C1458
T1463
C1464

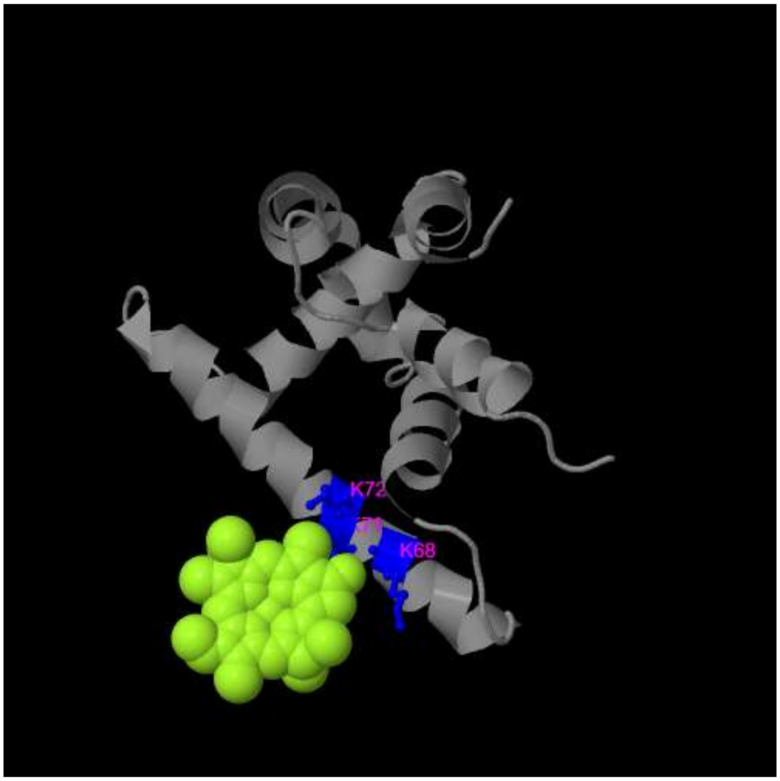
K72
K68

B. Tables

Supplemental Table S1. List of antigens selected and their putative function.

Antigen	Antigen Full name	Gene ID	Putative evidence	Reference
CELTOS	Cell-traversal protein for ookinetes and sporozoites	PF3D7_1216600	Associated with protection from clinical malaria	21
CSP	Circumsporozoite protein	PF3D7_0304600	Associated with protection from clinical malaria.	22
LSA1	Liver stage antigen 1	PF3D7_1036400	Associated with protection from clinical malaria.	23
LSA3	Liver stage antigen 3	PF3D7_0220000	Associated with protection from clinical malaria.	24
P36	6-cysteine protein (P36)	PF3D7_0404400	Gene knockout prevents parasite egress from liver	25
P52	6-cysteine protein (P52)	PF3D7_0404500	Gene knockout prevents parasite egress from liver	26
UIS3	Upregulated in infectious sporozoite	PF3D7_1302200	Gene knockout prevents parasite egress from liver	27
UIS4	Early transcribed membrane protein	PF3D7_1016900	Gene knockout prevents parasite egress from liver	28
SAP1	Sporozoite and liver stage asparagine-rich protein		Gene knockout abolish liver stage	29
SPECT2	sporozoite microneme protein essential for cell transversal	PF3D7_1147000	Essential for cell transversal	
		PF3D7_040870		30

Supplemental Table S2. Samples origin .

**Continent**	**Country**	**Number of Sequences**	***P. falciparum* endemicity**	**Collection year**
Africa	Burkina Faso	56	High	2008
	Cameroon	130	High	2013
	Guinea	123	High	2011
	Kenya	57	High	2007
	Malawi	150	High	2007-08, 2014-16
	Mali	99	High	2002, 2010
	Tanzania	68	High	2010, 2013
Asia	Cambodia	129	High	2009-11
	Myanmar	18	Low	2013
	Thailand	36	Low	2013-14
Oceania	Papua New Guinea	59	Low	2008-10
South America	Brazil	22	Low	2016
	Colombia	16	Low	2011
	French Guiana	36	Low	2009-2013
	Peru	11	Low	2011

Supplemental Table S3. **FST values of the malaria vaccine candidates by continents**. Continents are in columns and each Excel sheet represent a different malaria vaccine candidate.

Supplemental Table S4. **FST values of the malaria vaccine candidates by countries**. Countries are in columns and each Excel sheet represent a different malaria vaccine candidate.

Supplemental Table S5. **Haplotypes distribution by country.** Prevalence of ten of the most frequent haplotypes of each of the studied malaria vaccine candidate by country. Each column represents a country and haplotypes, and their respective frequencies are in row. Each Excel sheet represents a different malaria vaccine candidate.

Supplement Table S6. Putative, strong binding CD4+ and CD8+ T-cell epitopes in malaria vaccine candidates.

Antigen	Strong binding CD4+ epitope	Strong binding CD8+ epitope	Number of strong and medium binders	Average number of epitopes per 100 amino acid residues (AA) (Protein length, in AA)
CelTOS				
	MNALRRLPVICS	LPVICSFLVF	9	4.9 (182)
CSP				
	KLAILSVSSFLF<br>LAILSVSSFLFV<br>ENWYSLKKNSRS	SSFLF**V**EALF*	10	2.5 (397)
LSA1				
	TNFKSLLRNLGV<br>NFKSLLRNLGVS<br>QTNFKSLLRNLG<br>FKSLLRNLGVSE<br>NFKSLLRNLGVS<br>TNFKSLLRNLGV<br>ISFYFILVNLLI<br>SFYFILVNLLIF	KFIKSLFHIF*	16	1.4 (1162)
LSA3				
	None	ASYVVGFFTF*<br>SYVVGFFTFS*<br>PFYSFVFDIF*<br>KVKNFVKKYK<br>KVDKNNKVPK*<br>KTRKKAQRPK*<br>KVFAAPFISA*<br>KINKYFFLIK	25	1.6 (1558)
P36				
	IRYNKSRSSNLI<br>AIRYNKSRSSNL<br>KFVQLLKSIDIK<br>RYNKSRSSNLIA<br>FVQLLKSIDIKM<br>AIRYNKSRSSNL<br>EAIRYNKSRSSN<br>EEAIRYNKSRSS	KSKCETKIEK	20	5.3 (379)

	IRYNKSRSSNLI<br>SKFVQLLKSIDI			
P52				
	MCYHFTMKRKKL<br>HMCYHFTMKRKK<br>NLFGLSSSKYIL<br>QNLFGLSSSKYI<br>NININFVCSNVI<br>ININFVCSNVIQ<br>CYHFTMKRKKLF<br>YHFTMKRKKLFV<br>LFGLSSSKYILF<br>GTMIIYTKNINS<br>MIIYTKNINSLM<br>TMIIYTKNINSL<br>VGTMIIYTKNIN<br>FGLSSSKYILFN	KYILFNNFLI<br>ILFNNFLILF*<br>VYFIFLSFII*<br>KVKHIMRINI<br>RTRTFWQNLF<br>KLSRNHSFSS<br>NPSNCFHDVY<br>NPSNCFHDVY<br>NPSNCFHDVY<br>NPSNCFHDVY	39	8.15 (478)

Antigen	Strong binder CD4+ epitope	Strong binder CD8+ epitope	Number of Strong and medium binders	Average number of epitopes per 100 amino acid residues (AA) (Protein length, in AA)
SAP1				
	None	VKYFNKPIQF<br>YKYIQNIILF<br>YFMPKNDLNF<br>KYIQNIILFL<br>NYMPQNYYHI<br>RIFFSFFSYF<br>RFKLTCNFKF<br>KLKNFFLNYK<br>YTRAVWLLKK<br>MPKNDLNFIF<br>MPQNYYHINY	43	1.5 (2940)
SPECT2				
	KLRILKKHYYVV*<br>LRILKKHYYVVF*<br>MKLRILKKHYYV*<br>MKLRILKKHYYV*<br>KLRILKKHYYVV*<br>LRILKKHYYVVF*	LYFIGIGYNL<br>IYVLCVDTTI<br>KRSKKTFLVK<br>KVVMFGFSLK<br>RSKKTFLVKS<br>KKIKHSFNLA<br>YIPWDKTTAY	34	4 (842)
UIS3				
	KYHLQKVYANYL*<br>YHLQKVYANYLS*			

	MEYMQKFVQGLQ*<br>NMEYMQKFVQGL*<br>NNMEYMQKFVQG*<br>VNNMEYMQKFVQ*<br>LIYYSLIASGAI*<br>IYYSLIASGAIA*	None	9	3.9 (229)
UIS4				
	KQIIISTAAVAL*<br>QIIISTAAVALA*<br>RTLEKLLRKKQI*<br>DRTLEKLLRKKQ<br>LEKLLRKKQII*	None	4	3.7 (108)

Bold: Mutated AA residue relative to 3D7 allele.
* Have a mutation
Underline: Mutations likely influence peptide function
